# Porous Metal‐Organic Cages Based on Rigid Bicyclo[2.2.2]oct‐7‐ene Type Ligands: Synthesis, Structure, and Gas Uptake Properties

**DOI:** 10.1002/chem.202300732

**Published:** 2023-04-25

**Authors:** Beatriz Doñagueda Suso, Alexandre Legrand, Catherine Weetman, Alan R. Kennedy, Ashleigh J. Fletcher, Shuhei Furukawa, Gavin A. Craig

**Affiliations:** ^1^ Department of Pure and Applied Chemistry University of Strathclyde Glasgow G1 1XL UK; ^2^ Institute for Integrated Cell-Material Sciences (WPI-iCeMS) Kyoto University iCeMS Research Building Yoshida, Sakyo-ku Kyoto Japan; ^3^ Unité de Catalyse et Chimie du Solide (UCCS) Université de Lille CNRS Centrale Lille Université d'Artois UMR 8181 59000 Lille France; ^4^ Department of Chemical and Process Engineering University of Strathclyde Glasgow G1 1XJ UK; ^5^ Department of Synthetic Chemistry and Biological Chemistry Kyoto University iCeMS Research Building Yoshida, Sakyo-ku Kyoto Japan

**Keywords:** gas uptake, ligand design, metal-organic cages, porous materials, supramolecular chemistry

## Abstract

Three new ligands containing a bicyclo[2.2.2]oct‐7‐ene‐2,3,5,6‐tetracarboxydiimide unit have been used to assemble lantern‐type metal‐organic cages with the general formula [Cu_4_L_4_]. Functionalisation of the backbone of the ligands leads to distinct crystal packing motifs between the three cages, as observed with single‐crystal X‐ray diffraction. The three cages vary in their gas sorption behaviour, and the capacity of the materials for CO_2_ is found to depend on the activation conditions: softer activation conditions lead to superior uptake, and one of the cages displays the highest BET surface area found for lantern‐type cages so far.

## Graphical abstract

Ligands derived from bicyclo[2.2.2]oct‐7‐ene‐2,3,5,6‐tetracarboxydiimide units are found to provide a suitably rigid backbone for the synthesis of porous metal‐organic cages. Crystallographic studies show that the cages display a degree of flexibility in the solid state, while the activation protocols followed are a determining factor in the gas uptake capacity of the cages.

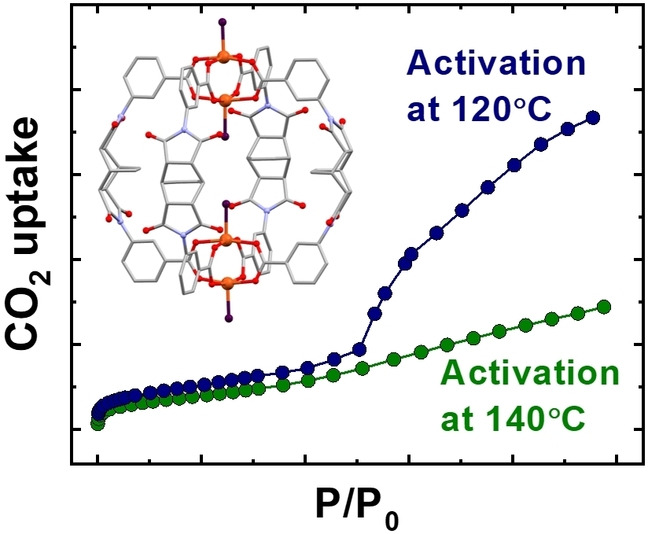

## Introduction

Metal‐organic polyhedra (MOPs) or Metal‐organic cages (MOCs) are molecules capable of displaying permanent porosity.^[1]^ To observe the intrinsic porosity of these cages in the solid state, neighbouring molecules should not block the pore windows upon desolvation, and the individual MOPs must be sufficiently rigid and thermally stable to retain a cavity. Predicting how the molecules will rearrange during activation is difficult,^[2]^ and the intermolecular control of extrinsic porosity remains a challenge for MOCs. The use of rigid ligands containing carboxylic acid groups that form metal paddlewheel units or higher nuclearity clusters is the most common approach to conserve the intrinsic pore of the cages.^[3]^ This approach was adopted from the use of secondary building units (SBUs) in metal‐organic frameworks (MOFs),^[4]^ as motifs such as the paddlewheel were found to offer greater thermal stability and a higher probability of enabling permanent porosity than monotopic N‐donor ligands.^[5]^ Designing the directional bonding of the carboxylates then determines the geometry of the MOP, where the largest family consists of cuboctahedral MOPs of the general formula [M_24_L_24_], where L is an isophthalate derivative and M_24_ represents twelve [M_2_] paddlewheel units.^[6]^

Lantern‐type MOPs are the lowest nuclearity metal‐organic cages studied for gas storage in the solid state. They have a general formula of [M_2_L_4_]^2+^ or [M_4_L_4_], although [M_2_L_4_]^2+^‐type cages, where M is typically Pd(II) or Pt(II), are rarely studied for permanent porosity.^[7]^ The majority of [M_4_L_4_]‐type cages feature derivatives of 3,3′‐((1,3‐phenylene)bis(ethyne‐2,1‐diyl))dibenzoic acid combined with [Cu_2_] or [Rh_2_] paddlewheels.^[8]^ Functionalisation of the phenylene ring in these systems has led to the observation of cooperative gas uptake in lantern‐type MOPs,^[9]^ while both the ligand backbone and metal nodes have been used for post‐synthetic assembly of the cage units into polymers.^[10]^ Based on the crystal structures reported for this cage architecture, they display the largest average pore size for porous metal‐organic lanterns, of 9.3(2) Å when measured across the pore between the internal metal ions of the paddlewheel units (see below). The smallest average pore size for lanterns is found for cages based on *meta*‐terphenyl derivatives (5.1(4) Å). Recent work has used ligand isomerism to access pore sizes lying in between these two extremes,^[11]^ as well as design of new pyrazolyl‐based ligands.^[12]^

Herein, we describe three new ligands based on bicyclo[2.2.2]oct‐7‐ene units. This moiety was recently used in the formation of porous organic cages prepared via condensation reactions with polyamines, with the rigidity of the unit sufficient to maintain the pore of the cage molecules.^[13]^ In our work, the bicyclo[2.2.2]oct‐7‐ene units provide the curvature needed for the lantern geometry of metal‐organic cages. The three examples of lantern‐type metal‐organic cages we detail are found to sustain elongation or compression along the inter‐paddlewheel axis, affecting the size of the cavity in the molecules. The packing of the molecules arises from the combination of the functional groups on the backbone of the cages with the role played by the solvent molecules coordinated to the exterior paddlewheel site of the Cu(II) ions. Gas sorption isotherms measured for N_2_ and CO_2_ uptake at 77 and 195 K show that one of the cages displays the highest BET surface area reported for lantern‐type MOPs.

## Results and Discussion

### Synthesis

**Figure 1:**
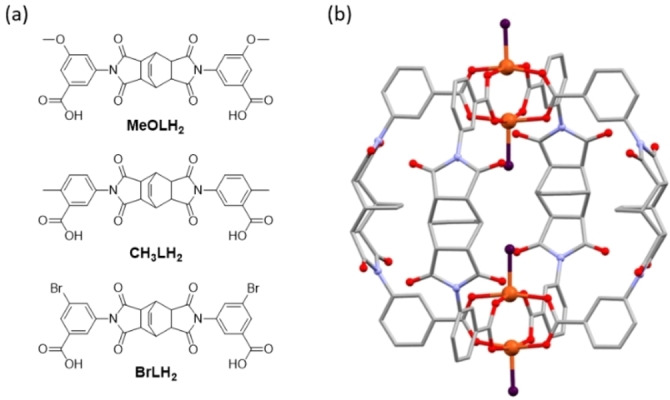
(a) Ligands used for the synthesis of the MOPs. (b) View of the generic MOP structure for the cages described in this work, based on the crystal structure data for **1‐DMA**; copper: orange; carbon: grey; nitrogen: light purple; oxygen: red; coordinated solvent molecules are represented by the dark purple spheres. Hydrogen atoms are omitted.

By reacting functionalised 3‐aminobenzoic acid derivatives with bicyclo[2.2.2]oct‐7‐ene‐2,3,5,6‐tetracarboxylic dianhydride in acetic acid, three new ligands, **MeOLH_2_**, **CH_3_LH_2_**, and **BrLH_2_**, were obtained in good yields (see Figure 1; full synthetic details are provided in the experimental section). The MOPs were synthesised by reaction of the ligands and salts of Cu(II) in a 1 : 1 ratio in dimethylacetamide (DMA) at 80 °C. Reaction of **MeOLH_2_** or **CH_3_LH_2_** and Cu(NO_3_)_2_ or Cu(OAc)_2_ ⋅ H_2_O, respectively, in DMA directly yielded single crystals of the MOPs [Cu_4_(MeOL)_4_(DMA)_2_(H_2_O)_2_]⋅13DMA ⋅ 2H_2_O (**1‐DMA**) and [Cu_4_(CH_3_L)_4_(DMA)_2_(H_2_O)_2_] ⋅ 11DMA ⋅ H_2_O (**2‐DMA**). Single crystals of the MOP [Cu_4_(BrL)_4_(H_2_O)_4_] ⋅ 15DMA ⋅ 2H_2_O (**3‐DMA**) were obtained after heating the reaction, cooling to room temperature, and then layering the resultant solution with MeOH. Both **1‐DMA** and **3‐DMA** could be obtained in higher yields as polycrystalline solids through use of Cu(OAc)_2_ ⋅ H_2_O as the metal salt, and confirmed as the same phase as the single‐crystal structures by powder X‐ray diffraction (PXRD) (see Figures S8–S10). Once formed, the cages were found to be insoluble in organic solvents, including DMA and dimethylformamide. The solvent molecules coordinated to the cage molecules were identified using the single crystal X‐ray diffraction (SXRD) data, while the non‐coordinated solvent content was determined using a combination of ^1^H NMR digestion experiments (Figures S11–S13), thermogravimetric analysis (TGA, Figures S14–S16), and use of the SQUEEZE algorithm that accounts for disordered electron density in the crystal structure.^[14]^ The final formula provided originates from the X‐ray data.

### Single crystal structures

**Figure 2:**
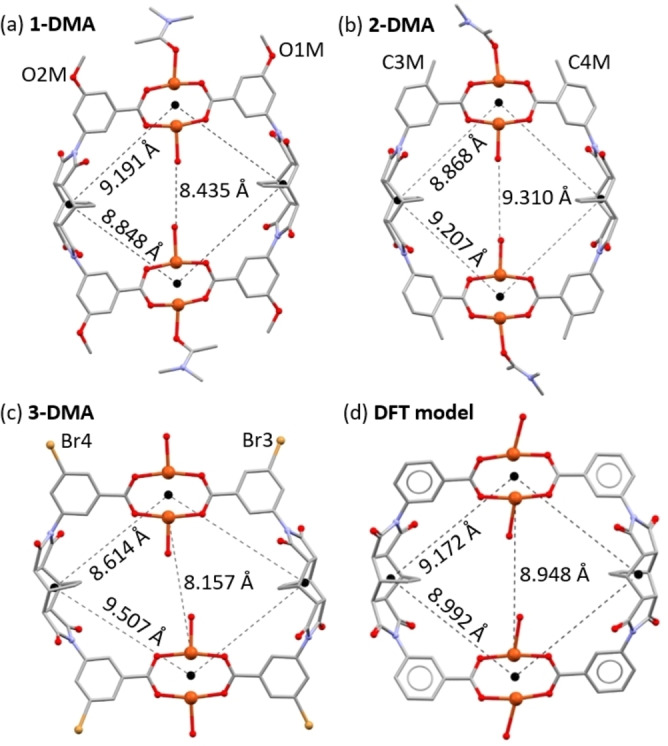
View of the distortion in the cage molecules of (a) **1‐DMA**, (b) **2‐DMA**, and (c) **3‐DMA**, as measured through the unequal distances between the paddlewheel units and a centroid at the midpoint of the bicyclo[2.2.2]oct‐7‐ene moiety. The distance across the pore between the internal Cu(II) ions is also shown. The structure obtained from DFT calculations is presented in (d).

The SXRD data show that all three MOPs are neutral cages composed of two Cu(II) paddlewheel units connected through four deprotonated ligands. **1‐DMA** and **2‐DMA** crystallise in the monoclinic space groups *C*2/*c* and *P*2_1_/*n*, respectively, while **3‐DMA** crystallises in the triclinic space group *P*‐1 (Table S1 contains crystallographic parameters). In all of the structures, the centre of the intrinsic pore lies on an inversion centre, and the axial sites of the internal Cu(II) ions are occupied by water molecules. To form the lantern‐type geometry of the MOPs, there is a rotation of the benzoate rings around the N−C bond in the ligands that leaves the substituents (methoxy‐, methyl, and bromo‐ for **MeOL^2−^**, **CH_3_L^2−^**, and **BrL^2−^**, respectively) on the exterior of the MOP backbone, pointing away from the internal cavity. Therefore, the internal cavity of all three MOPs has the same chemical structure. However, the cages display large differences in their metric parameters. The cage in **2‐DMA** is relatively elongated (Figure 2), so that the distance between the internal Cu(II) ion and its symmetry equivalent on the other side of the cavity measures 9.310(1) Å, which contrasts with **1‐DMA** (8.435(1) Å), and the relatively flattened cage in **3‐DMA** (8.157(1) Å). These distances lie above and below the distance found for the DFT calculated structure based on a non‐functionalised ligand backbone, which gave a distance across the pore of 8.948 Å. This elongation or compression of the cages does not lead to a uniform distortion of the molecular geometry. For each MOP, a parallelogram can be defined by the connection of a pair of centroids at the midpoints of the Cu(II) paddlewheels and centroids at the midpoint of the bicyclo[2.2.2]oct‐7‐ene units (Figure 2, and Figures S17–S20). Of the three cages reported here, **3‐DMA** shows the largest difference between the two sides of the parallelogram of 0.893 Å (9.507 vs. 8.614 Å).

**Figure 3:**
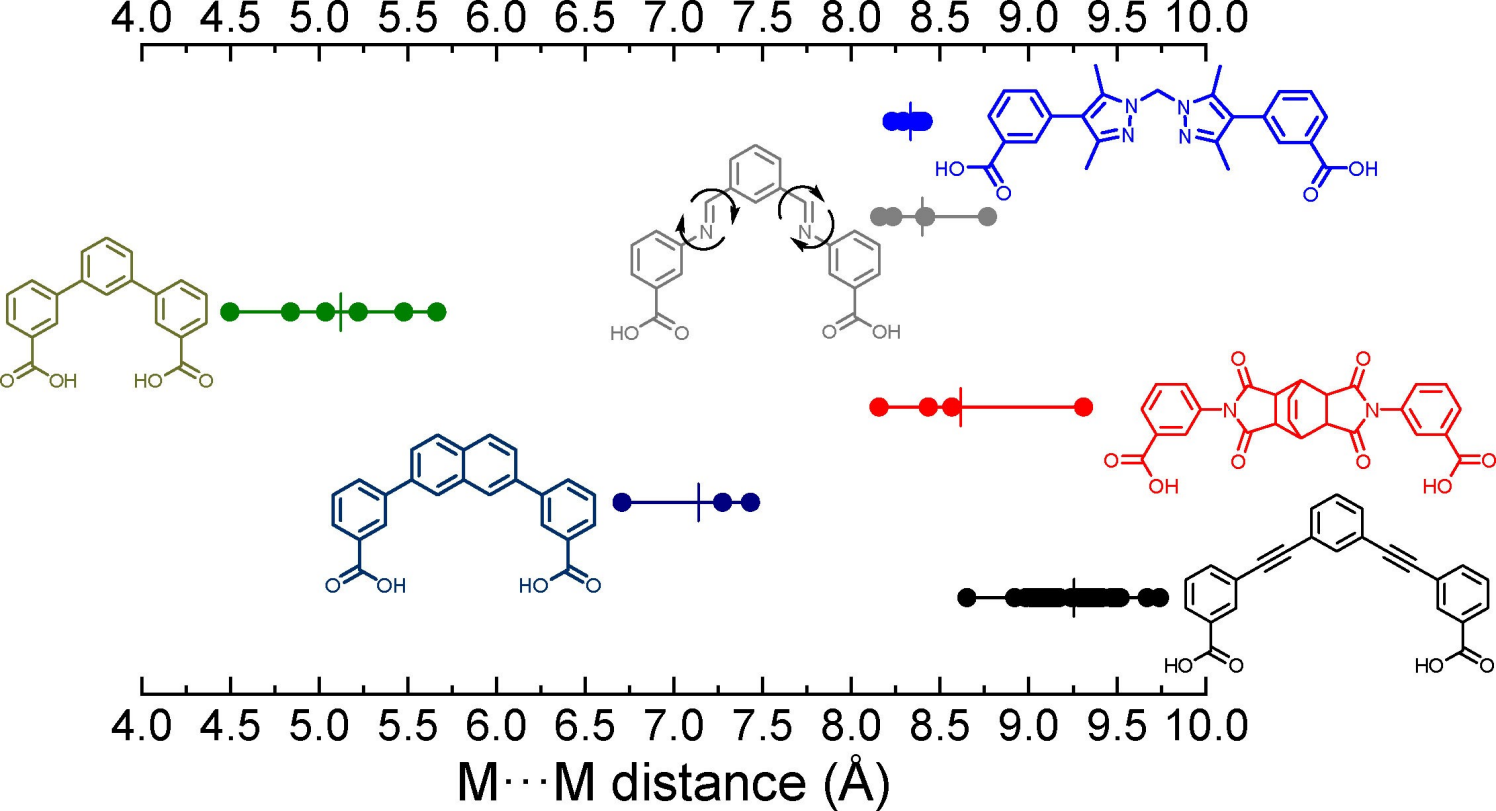
A plot of the measured pore sizes in lantern‐type MOPs, defined as the distance across the pore between the internal metal ion site of the paddlewheel. Each full circle represents the distance in Angstroms as measured in the SXRD data found in the CSD, and the vertical line denotes the mean value for that family of cages. Only the generic backbones are shown, without functionalisation; full details are compiled in the Supporting Information.

We analysed the structural variation in the pore sizes found in porous lantern‐type MOPs, as measured by the distance across the cavity of the cage between the internal metal ions of the paddlewheel units. For this, we focussed on families of compounds that had been used for study of their gas sorption properties, and identified six generic types (Figure 3; full details of the functional groups and Cambridge Structural Database refcodes for these molecules are provided in the Supporting Information). Of these, four families might be anticipated to be relatively rigid and show limited variation in the distance across the pores: those based on 3,3′‐((1,3‐phenylene)bis(ethyne‐2,1‐diyl))dibenzoic acid‐type ligands (black in Figure 3); *meta*‐terphenyl ligand derivatives (green in Figure 3); naphthalene‐derivatives (navy blue in Figure 3); and the cages reported here based on bicyclo[2.2.2]oct‐7‐ene moieties. However, depending on metallic composition and functionalisation of the ligand backbone, each of these families presents a range of cavity sizes. The most numerous family consists of cages derived from 3,3′‐((1,3‐phenylene)bis(ethyne‐2,1‐diyl))dibenzoic acid‐type ligands. The largest pore size observed for these molecules is found for cages containing [Mo_2_] paddlewheel units (XUQJOK, 9.738 Å; XUQJIE, 9.668 Å).^[8f]^ The smallest pores for lantern‐type MOPs are found when *meta*‐terphenyl ligand derivatives are used – Taggart and co‐workers observed a pore distance of 4.497 Å for [Cu_4_(pdb)_4_] (pdb=pyridinedibenzoate), which represents the smallest pore size observed so far for this type of molecule.^[15]^ Surprisingly, there is little correlation between the cavity size observed and the solvent molecule coordinated to the inner site of the MOP. As highlighted above in the case of **1‐DMA**, **2‐DMA**, and **3‐DMA**, in which there is a maximum difference of 1.153 Å between **2‐DMA** and **3‐DMA**, the cavities of the MOPs contain coordinated water molecules, rather than the bulkier DMA solvents used in the synthesis. Therefore, it is difficult to offer design rules for these structural variations as they depend on composition, on the electronic and steric effects that arise from functionalisation, and particularly on crystal packing.

**Figure 4:**
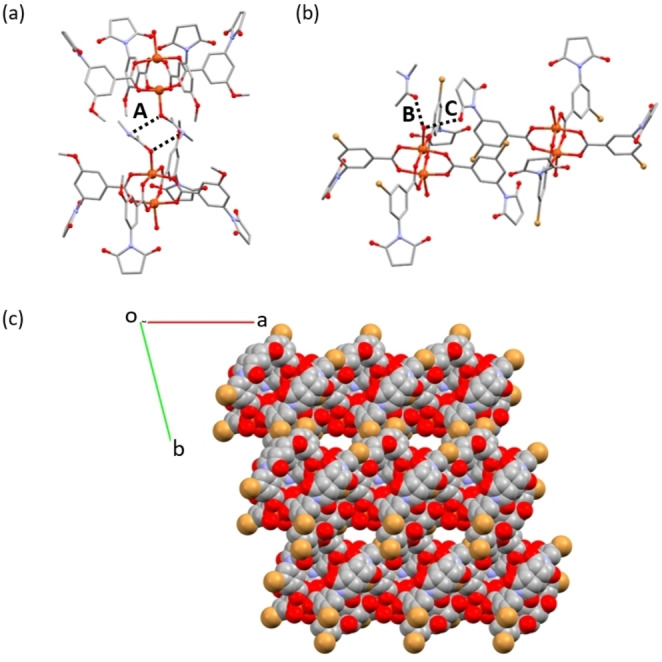
(a) View of the interaction (A) between DMA molecules coordinated to the axial sites of the paddlewheel of **1‐DMA**, with the MOPs belonging to adjacent layers of the crystal structure. (b) View of the hydrogen bonding motif formed by the H_2_O molecule coordinated to the axial site of the paddlewheel in **3‐DMA**, which interacts with a molecule of DMA from the crystal structure (B) and the oxygen atom of the dicarboximide ring on an adjacent MOP (C). (c) View along the *c*‐axis of the crystal structure of **3‐DMA**, showing the channels that are occupied by DMA molecules of solvation, which have been omitted for clarity.

The crystal packing for **1‐DMA** and **2‐DMA** is similar in that they consist of stacked sheets of MOPs. In **1‐DMA** these sheets lie in the *bc* plane (Figure S21). The most important interactions in these sheets are O3⋅⋅⋅π contacts, where O3 is an oxygen atom from the donor carbonyl group displaying a short interaction (2.813(2) Å) with the centroid of an acceptor dicarboximide ring on a neighbouring MOP. The sheets stack with the interaction between layers mediated by the coordinated DMA molecules of the cage (Figure 4(a) and Figure S22). In **2‐DMA**, sheets of MOPs form in the (1 0 −1) plane of the structure, through O⋅⋅⋅π interactions (Figure S23). These sheets stack with the axially coordinated DMA molecules of one layer slotting into the gaps between MOPs of a neighbouring layer (Figures S24 and S25). In both **1‐DMA** and **2‐DMA**, the packing of cage molecules is relatively tight, and doesn't lead to noticeable ordered channels in the crystal structure. In **3‐DMA** the external axial position of the Cu(II) paddlewheel is occupied by a water molecule, rather than DMA. These water molecules induce the formation of hydrogen bonding motifs where one hydrogen bond is formed with a non‐coordinated DMA molecule (O100−O1W), and a second is formed with the carbonyl group of a dicarboximide ring on a neighbouring MOP (O1W−O2). The molecular packing leaves channels between the cages along the c‐axis of the crystal structure, which are occupied by DMA solvent molecules (Figure 4).

### Solvent exchange processes

**Figure 5:**
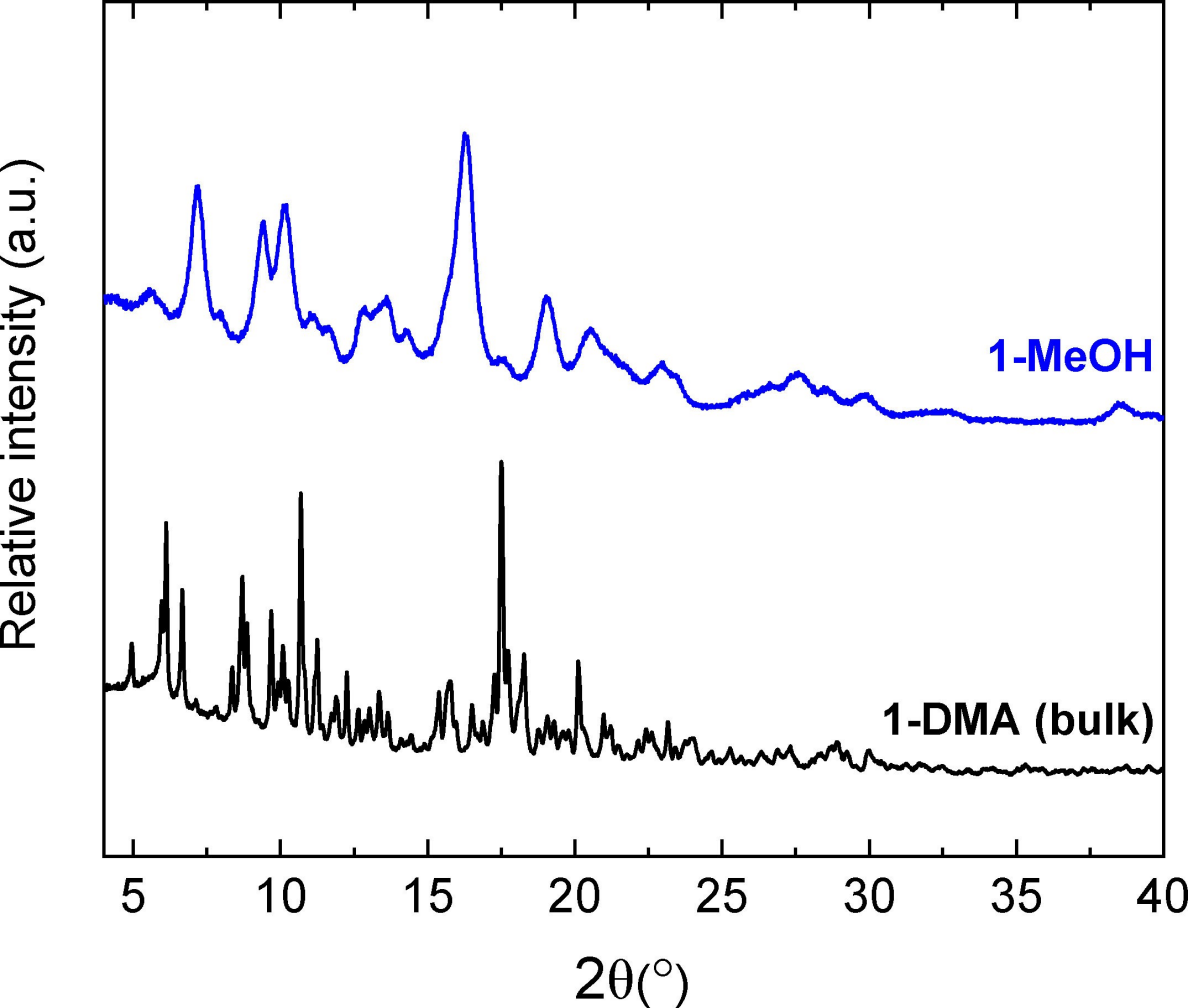
Powder X‐ray diffraction data for the phases **1‐DMA** and **1‐MeOH**, illustrating the loss of crystallinity upon treatment with MeOH.

The bulk samples of **1‐DMA**, **2‐DMA**, and **3‐DMA** were soaked in MeOH to exchange the more volatile alcohol for the DMA molecules in the structure, yielding the new phases **1‐MeOH**, **2‐MeOH**, and **3‐MeOH**. This process was monitored using PXRD, TGA, IR spectroscopy (Figures S26–S28), and ^1^H NMR spectroscopy of the new phases. The ^1^H NMR spectra collected on acid‐digested samples of these new phases show the disappearance of the peaks arising from DMA (Figure S29‐S31), and the presence of a singlet at 3.11 ppm due to MeOH. The TGA measurements show that the exchange process has a negligible effect on the overall stability of the cages, with the onset of decomposition occurring close to 300 °C for all of the cages, both before and after solvent exchange (Figures S14–S16). Based on the TGA data, the proposed formulae for these new phases are [Cu_4_(MeOL)_4_(H_2_O)_2_(MeOH)_2_] ⋅ 5MeOH (**1‐MeOH**), [Cu_4_(CH_3_L)_4_(H_2_O)_2_(MeOH)_2_] ⋅ 6MeOH (**2‐MeOH**), and [Cu_4_(BrL)_4_(H_2_O)_2_(MeOH)_2_] ⋅ 3MeOH (**3‐MeOH**). The PXRD patterns collected on these new phases (Figures S8‐S10) show that this solvent exchange process alters the crystal packing for all three cages, which is to be expected given the role of the solvents in the crystal packing described above. This is commonly found for MOPs, where often this process also causes a loss of crystallinity, and the three phases **1‐MeOH**, **2‐MeOH**, and **3‐MeOH** could only be obtained as polycrystalline powders, rather than single crystals. Of these, **1‐MeOH** shows the biggest loss of crystallinity, and a significant broadening of the diffraction peaks (Figure 5).

### Gas sorption measurements

**Figure 6:**
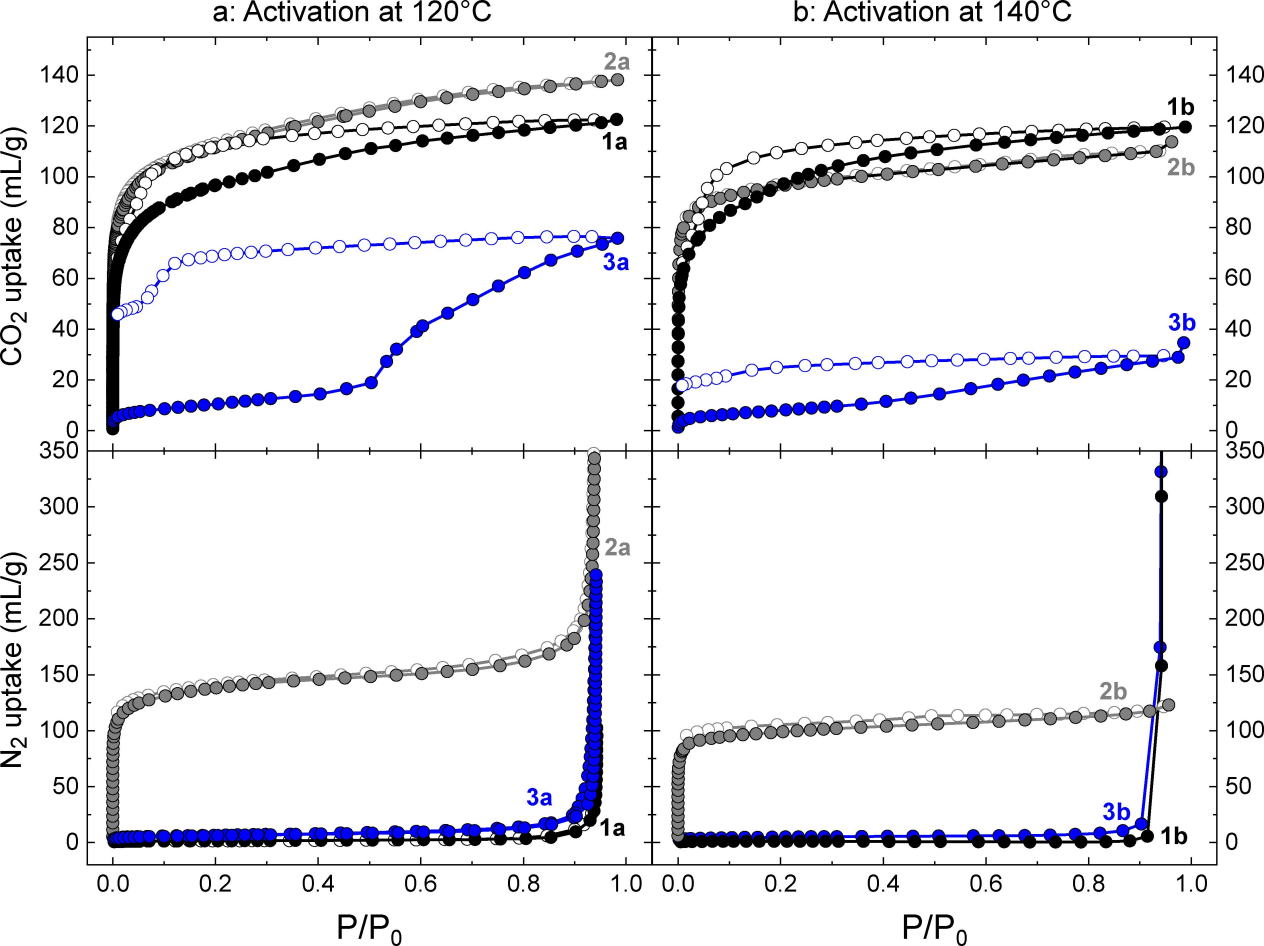
Gas sorption isotherms for compounds **1 a** and **1 b**, **2 a** and **2 b**, and **3 a** and **3 b**. Isotherms on the left hand side were measured after activation under vacuum at 120 °C, while the isotherms on the right hand side were measured after activation under vacuum at 140 °C. (top) CO_2_ uptake measured at −78.15 °C, and (bottom) N_2_ uptake measured at −196.15 °C. Full circles represent adsorption and empty circles represent desorption. The desorption branches of the N_2_ uptake for compounds **1 b** and **3 b** were not measured.

The gas sorption properties of all three cages were measured for both N_2_ and CO_2_ at 77 and 195 K, respectively (Figure 6). The cages **1‐MeOH**, **2‐MeOH**, and **3‐MeOH** were activated by heating under vacuum at 120 °C, to yield the samples **1 a**, **2 a**, and **3 a**, respectively. Cages **1 a** and **3 a** show low uptake of N_2_, displaying Type III isotherms^[16]^ with a steep increase observed for *P*/*P*_0_ >0.9 due to bulk condensation. On the other hand, **2 a** displays a sharp uptake of N_2_ in the region 0.02 > *P*/*P*_0_, reaching approximately 116 mL/g. Beyond this pressure, the uptake increases more gradually to 182 mL/g at *P*/*P*_0_=0.90. The BET surface area for **2 a** calculated using BETSI (Brunauer, Emmett and Teller Surface Identification) is 521 m^2^/g (Figure S32).^[17]^ Experimental determination of surface areas for lantern‐type MOPs is rare, which we suggest is due to the often low observed uptake of N_2_ by these cages. Until now, the highest reported surface area was of 455 m^2^/g, for a lantern‐type cage published by Bloch.^[18]^ Measurement of the CO_2_ sorption isotherms shows a Type I isotherm for the uptake by **2 a**, which attains a maximum uptake of 138 mL/g for *P*/*P*_0_≈0.98. **1 a** also displays an adsorption isotherm consistent with Type I (122 mL/g for *P*/*P*_0_≈0.98), but the desorption branch diverges from the adsorption branch upon decreasing pressure with an inflection point at *P*/*P*_0_≈0.05 (89 mL/g). **3 a** presents a structured hysteresis loop, with a step in the uptake occurring at *P*/*P*_0_≈0.50 (19 mL/g), reaching a maximum of 75 mL/g at *P*/*P*_0_≈0.98. The desorption branch shows a gradual decrease in the volume of CO_2_ adsorbed, reaching 66 mL/g at *P*/*P*_0_≈0.12, before decreasing more rapidly and reaching a minimum of 46 mL/g at *P*/*P*_0_≈0.01. Structured hysteresis loops of this sort have been observed in metal‐organic cages before,[^3e^, ^8b^, ^9^] where they may be associated with structural changes being induced by incorporation of the gas molecules. There are also examples of hysteresis due to enhanced interactions between cages and the sorbates.[^5b^, ^19^] However, as in references 5b and 19, the exact structural origin of the step and hysteresis observed in **3 a** is unclear. Given the differences in the functionalisation of the ligands, we speculate that there may be enhanced interactions between CO_2_ molecules and the bromo‐functionalised ligand of **3 a** in comparison to the cages **1 a** and **2 a**. Halogen bonding interactions with CO_2_ have been proposed as leading to pseudo‐gate opening behaviour in MOFs,^[20]^ but the role of those interactions is contested.^[21]^ The lack of structural information for MOPs in their activated state is common in the field, and represents a challenge for understanding their structure‐property relationships, particularly with gas sorption behaviour. The post‐sorption analysis of **1 a**, **2 a**, and **3 a** using IR spectroscopy and PXRD showed that the materials retain the packing associated with the MeOH solvate phases **1‐MeOH**, **2‐MeOH**, and **3‐MeOH**, suggesting that the materials do not decompose under activation (Figures S33–S38), although there is an appreciable loss in crystallinity. The post‐sorption ^1^H NMR digestion experiments for the samples revealed that the activation process at 120 °C did not fully remove MeOH from the samples (Figures S39–S44). For **1 a**, **2 a**, and **3 a**, remaining MeOH content was found to be equivalent to 0.8 MeOH/MOP, 0.08 MeOH/MOP, and 1.4 MeOH/MOP, respectively. Considering the TGA profiles for **1‐MeOH**, **2‐MeOH**, and **3‐MeOH**, we propose that the MeOH found in the samples corresponds to MeOH molecules coordinated to the paddlewheel motifs of the MOPs, as non‐coordinated MeOH molecules should be removed under these activation conditions. To achieve a greater degree of activation, fresh samples of **1‐MeOH**, **2‐MeOH** and **3‐MeOH** were activated at 140 °C under vacuum, yielding the cages **1 b**, **2 b**, and **3 b**. The post‐sorption analysis showed that the MeOH content was lower for **1 b** at 0.4 MeOH/MOP; slightly lower for **2 b** at 0.03 MeOH/MOP; and the same for **3 b** at 1.4 MeOH/MOP. The effect of this higher temperature protocol on the gas sorption properties of the materials is shown in Figure 6. For all three cages, a reduction in the capacity of the materials for CO_2_ is observed. **3 b** shows the largest reduction in capacity, dropping by more than half to 28 mL/g at *P*/*P*_0_≈0.98, compared to 75 mL/g observed for **3 a**. The BET surface area for **2 b** is also found to be lower, at 382 m^2^/g (Figure S45). Inspection of the PXRD data for the samples of **1 a** and **1 b** subsequent to gas sorption measurements show that the MOPs recover the crystal packing associated with the phase **1‐MeOH**, although **1 b**, which was subjected to harsher activation conditions, shows a greater degree of broadening in the diffraction peaks than **1 a**. This is also the case for **2 a** and **2 b**, particularly in the region of 2*θ* between 7.5 and 12.5° (Figure S37). For **3 a** and **3 b**, both phases are significantly less crystalline than the parent phase **3‐MeOH**. It should be noted that the diffraction data are collected after the samples have been removed from the gas sorption apparatus – previous work using in situ techniques has shown that the activated phases of MOPs can display distinct packing to parent solvates.^[9]^ We hypothesise this may also be the case for **3 a** and **3 b**: upon exposure to air, the open metal sites of the paddlewheels capture moisture, resulting in both samples recovering a packing similar to **3‐MeOH** in the subsequent PXRD measurements. These results highlight the sensitivity of the gas uptake properties of metal‐organic cages to activation conditions,^[22]^ especially in the case of **3 a**/**3 b**, where the molecular composition is the same after both degassing protocols but where the reduction in CO_2_ uptake is largest.

## Conclusion

In conclusion, we have demonstrated the potential for a new type of ligand in the synthesis of lantern‐type metal‐organic cages with permanent porosity. Cages **1–3** illustrate that the bicyclo[2.2.2]oct‐7‐ene‐2,3,5,6‐tetracarboxydiimide backbone is capable of showing flexibility in the size of the pore environment, as evidenced by the crystal structures of the materials. What is less clear is the relative effect of the electron withdrawing/donating character of the ligand substituents in contributing to these structural differences, or whether they are caused by solid state packing effects. The gas sorption measurements for these cages show that **2** has the highest surface area observed so far for lantern‐type cages. Meanwhile, cage **3** shows potentially cooperative gas sorption phenomena when the metal paddlewheel is not fully desolvated under relatively soft activation conditions. Although this type of solvent driven continuous breathing behaviour has been seen in framework materials,^[23]^ this is the first time that it has been observed for metal‐organic cages, and suggests that a variety of diverse gas sorption behaviours could be observed in cages by using this approach as a tool.

## Experimental Section

Solvents and starting reagents were used as purchased from Sigma Aldrich, Alfa Aesar, Fisher or Acros Organics without further purification. NMR and IR spectra detailed below are provided in the Supporting Information. The synthetic procedure for the ligands was based on a previously reported protocol.^[24]^

**MeOLH_2_**: Bicyclo[2.2.2]oct‐7‐ene‐2,3,5,6‐tetracarboxylic acid dianhydride (0.368 g, 1.486 mmol) was mixed with 3‐amino‐5‐methoxybenzoic acid (0.497 g, 2.973 mmol) in 15 mL of acetic acid. The resulting mixture was refluxed overnight forming a white suspension. This suspension was filtered and washed with water (40 mL), then ethanol (15 mL), and dried in air giving 0.662 g of white powder (Yield 81.5 %). ^1^H NMR (DMSO‐d_6_, 400 MHz, 25 °C) *δ* (ppm): 13.27 (s, 2H), 7.48 (dd, 2H, ^4^ *J*=2.5 Hz, 1.4 Hz), 7.31 (dd, 2H, ^4^ *J*=1.9 Hz, 1.3 Hz), 6.98 (dd, 2H, ^4^ *J*=2.5 Hz, 1.9 Hz), 6.36 (m, 2H), 3.82 (s, 6H), 3.53 (s, 2H), 3.43 (s, 4H). ^13^C NMR (DMSO‐d_6_, 400 MHz, 25 °C) *δ* (ppm): 176.4, 166.2, 159.4, 133.2, 132.6, 131.1, 119.8, 117.4, 113.8, 55.72, 42.48, 33.9. IR(cm^−1^): 1730, 1700, 1605, 1396, 1300, 1190, 1050, 891, 777, 687.

**CH_3_LH_2_**: Bicyclo[2.2.2]oct‐7‐ene‐2,3,5,6‐tetracarboxylic acid dianhydride (0.363 g, 1.486 mmol) was mixed with 5‐amino‐2‐methylbenzoic acid (0.451 g, 2.973 mmol) in 15 mL of acetic acid. The mixture was refluxed overnight giving a white suspension. This suspension was filtered and washed with water (40 mL) and ethanol (20 mL), resulting in 0.623 g of a white powder (Yield 82.8 %). ^1^H NMR (DMSO‐d_6_, 400 MHz, 25 °C) *δ* (ppm): 13.07 (s, 2H), 7.60 (d, 2H, ^4^ *J*=2.3 Hz), 7.40 (d, 2H, ^3^ *J*=8.3 Hz), 7.22 (dd, 2H, ^3^ *J*=8.3 Hz, ^4^ *J*=2.3 Hz), 6.33 (m, 2H), 3.52 (s, 2H), 3.42(s, 4H), 2.54 (s, 6H). ^13^C NMR (DMSO‐d_6_, 400 MHz, 25 °C) *δ* (ppm): 176.6, 167.7, 139.5, 132.1, 131.0, 130.9, 129.8,129.7, 128.3, 42.50, 33.89, 20.93. IR (cm^−1^):1718, 1503, 1390, 1186, 1150, 1090, 789, 675, 590, 550.

**BrLH_2_**: Bicyclo[2.2.2]oct‐7‐ene‐2,3,5,6‐tetracarboxylic acid dianhydride (0.292 g, 1.18 mmol) was mixed with 3‐amino‐5‐bromobenzoic acid (0.510 g, 2.36 mmol) in 15 mL of acetic acid. The mixture was refluxed overnight giving a white suspension. This suspension was filtered and washed with water (40 mL) and ethanol (20 mL), resulting in 0.577 g of a white powder (Yield 75.9 %). ^1^H NMR (DMSO‐d_6_, 400 MHz, 25 °C) *δ* (ppm): 13.60 (s, 2H), 8.07 (dd, 2H, ^4^ *J*=2.0 Hz, 1.3 Hz), 7.75 (dd, 2H, ^4^ *J*=1.8 Hz, 1.3 Hz), 7.67 (dd, 2H, ^4^ *J*=2.0 Hz, 1.7 Hz), 6.37 (m, 2H), 3.54 (s, 2H), 3.46(s, 4H). ^13^C NMR (DMSO‐d_6_, 400 MHz, 25 °C) *δ* (ppm):176.2, 165.1, 133.6, 133.5, 133.3, 131.6, 131.1,126.5, 121.3, 42.5, 33.9. IR (cm^−1^): 1705, 1581, 1463, 1373, 1286, 1245, 1180, 1082, 885, 791, 673, 625.

**[Cu_4_(MeOL)_4_(DMA)_2_(H_2_O)_2_]⋅13DMA ⋅ 2H_2_O (1‐DMA)**: A solution of **MeOLH_2_** (0.203 g, 0.3659 mmol) in DMA (2.5 mL) was added to a solution of Cu(OAc)_2_ ⋅ H_2_O (0.075 g, 0.3659 mmol) in DMA (2.5 mL). The resulting blue suspension was left to stand in an oven at 80 °C overnight giving a dark blue powder (Yield 372 mg). The resulting complex was characterized via IR spectroscopy, PXRD, TGA, and NMR spectroscopy of an acid digested sample. 285 mg of this bulk powder was soaked in MeOH and the supernatant was exchanged with fresh MeOH twice per day for 4 days giving 202 mg of a dark blue powder (**1‐MeOH**). For the formation of crystals suitable for single crystal X‐ray diffraction, a solution of **MeOLH_2_** (0.914 mmol, 49 mg) in DMA (1.25 mL) was mixed with a solution of Cu(NO_3_)_2_ (0.914 mmol, 23 mg) in DMA (1.25 mL). The resulting solution was placed in oven at 80 °C overnight giving light blue block crystals. Yield: 9 mg.

**[Cu_4_(CH_3_L)_4_(DMA)_2_(H_2_O)_2_] ⋅ 11DMA ⋅ 1H_2_O (2‐DMA)**: A solution of **CH_3_LH_2_** (0.188 g, 0.3659 mmol) in DMA (2.5 mL) was added to a DMA solution (2.5 mL) of Cu(OAc)_2_ ⋅ H_2_O (0.074 g, 0.3659 mmol). The resulting blue solution was left to stand in an oven at 80 °C overnight giving block crystals suitable for single crystal X‐ray diffraction (Yield 273 mg). The complex was characterized via IR spectroscopy, PXRD, TGA, and NMR spectroscopy of an acid digested sample. 195 mg of these crystals were solvent exchanged with fresh MeOH twice per day for 4 days giving 156 mg of a dark blue solid (**2‐MeOH**).

**[Cu_4_(BrL)_4_(H_2_O)_4_] ⋅ 15DMA ⋅ 2H_2_O (3‐DMA)**: A solution of BrLH_2_ (0.200 g, 0.31 mmol) in DMA (4.25 mL) was added to Cu(OAc)_2_ ⋅ H_2_O (0.063 g, 0.31 mmol) in DMA (4.25 mL). The resulting suspension was heated in an oven at 80 °C overnight giving a dark blue powder (Yield 217 mg). 192 mg of this bulk powder was solvent exchanged with MeOH twice per day for 4 days giving 160 mg of a light blue powder (**3‐MeOH**). The complex was characterized via IR spectroscopy, PXRD, TGA, and NMR spectroscopy of an acid digested sample. For the formation of single crystals, a solution of BrLH_2_ (0.0366 mmol, 20 mg) in DMA (0.5 mL) was mixed with a solution of Cu(NO_3_)_2_ (0.0366 mmol, 9 mg) in DMA (0.5 mL). The resulting solution was placed in oven at 80 °C overnight giving a suspension with a negligible quantity of a very fine powder. After centrifugation, the supernatant was layered with MeOH giving light blue block crystals within hours. Yield: 3 mg.

**Computational details**: All quantum chemical calculations were carried out using the Gaussian16 package.^[25]^ Geometry optimisation was performed using the B3LYP functional along with the 6‐31G(d) basis set.^[26]^ Initial geometry was based on the experimental single crystal data of compound **2‐DMA**, with the methyl group replaced with H and coordinated solvent replaced with H_2_O in the interest of computational resources and for model purposes. Each stationary point was identified by a subsequent frequency calculation as minimum (Number of imaginary frequencies NIMAG: 0). Cartesian coordinates are provided in the Supporting Information.

**Physical characterization**: ^1^H and ^13^C NMR spectra were measured with a Bruker AVANCE 400 NMR spectrometer at 25 °C operating at 400.13 MHz for ^1^H and 100.62 MHz for ^13^C. For acid digestion of the complexes, ca. 15 mg of the complex were suspended in DMSO‐d_6_ and 40 μL of DCL solution was added. The mixture was left to stand for 3 h at RT, resulting in a yellow solution suitable for NMR measurements. Infra‐red spectra were collected using a Thermo Scientific spectrometer model NICOLET iS5 using 64 scans and a resolution of 4 cm^−1^. Thermogravimetric analyses (TGA) were performed with a NETZSCH STA 449 F1 Jupiter under N_2_ using an isotherm for 10 min at 30 °C before heating up to 500 °C at a rate of 10 °C min^−1^. Powder X‐ray diffraction (PXRD) data were collected in a flat plate configuration using a Bruker D8 Discover diffractometer equipped with Cu K_α_ source (*λ*=1.54056 Å). The samples **1 a**, **2 a**, and **3 a** for gas sorption were activated in situ by heating the corresponding samples of **1‐MeOH**, **2‐MeOH**, and **3‐MeOH** under vacuum at 120 °C for 16 h, before measurement of the isotherms at 77 K (N_2_) and 195 K (CO_2_) using a BELSORP‐max volumetric adsorption instrument from BEL Japan, Inc. The temperature of the samples was controlled using a cryostat. Similarly, the samples of **1 b**, **2 b**, and **3 b** were obtained from heating fresh samples of **1‐MeOH**, **2‐MeOH**, and **3‐MeOH** under vacuum at 140 °C for 16 h. Post‐sorption measurement of IR spectra was performed on neat samples using a Jasco FT/IR‐6100 spectrometer. Post‐sorption measurement of NMR spectra were measured with a Bruker Ultrashield 500 plus (500 MHz) spectrometer at 25 °C. Post‐sorption PXRD data were collected using a Rigaku SmartLab diffractometer equipped with Cu Kα radiation (*λ*=1.54056 Å). Crystallographic data were measured using a Rigaku model XtaLAB Synergy‐i diffractometer equipped with a Hybrid Pixel Array Detector and using Cu Kα radiation (*λ*=1.54184 Å). All structures were solved with the SHELXT program using intrinsic phasing, and refined with ShelXL using least squares minimization (Table S1),^[27]^ within the program Olex 2–1.5.^[28]^ The SQUEEZE algorithm^[14]^ in PLATON^[29]^ was used to account for areas of where disordered solvent molecules could not be sensibly modelled.

For **1‐DMA**, all non‐hydrogen atoms on the skeleton of the MOP and solvent molecules were refined anisotropically. Hydrogen atoms were included in riding modes. Several parts of the structure were refined as disordered. Each disordered group was refined over two sites and each had appropriate constraints and restraints applied to ensure that normal geometry and normal displacement behaviour was approximated. The methoxy‐group containing O4ma, O4mb, C4ma and C4mb was modelled as being split over two positions with relative occupancy of 0.57 : 0.43. The DMA solvent molecules coordinated to outer axial sites of the paddlewheel formed by O1D, O1Z, N1D, N1Z, C1D, C1Z, C2D, C2Z, C3D, C3Z, C4D and C4Z were modelled as disordered in two positions with a relative occupancy of 0.31 : 0.69. DMA molecule formed by N104, N901 O105, O904, C100, C101, C103, C900, C902, C903, and C905 was modelled into two positions with a relative occupancy of 0.60 : 0.40. DMA molecule in the void containing N116, N801, O117, O804, C112, C113, C114, C115, C800, C802, C803 and C805 was modelled into two positions with a relative occupancy of 0.76 : 0.24. SQUEEZE was used to account for the remaining electron density, finding two different solvent voids: one with a volume of 1300 Å^3^ per unit cell containing 392 electrons (or 98 electrons per MOP). Two DMA molecules (96 electrons) can approximately account for this. The other solvent void calculated has a volume of 3784 Å^3^ per unit cell with 1060 electrons (or 265 per MOP) which corresponds approximately to five DMA molecules (240 electrons) and two water molecules (20 electrons).

In compound **2‐DMA** all non‐hydrogen atoms in the skeleton of the MOP were refined anisotropically, including the coordinated solvent molecules DMA and H_2_O. Hydrogen atoms were included in riding modes. SQUEEZE was used as a solvent mask to account for the disordered electron density in the void space of the structure, calculating a solvent void volume of 3834 Å^3^ in the unit with 1081 electrons (or 540 per MOP) that corresponds approximately to eleven DMA molecules (528 electrons) and one water molecule (10 electrons).

In compound **3‐DMA** all of the non‐hydrogen atoms in the skeleton were refined anisotropicaly including the water molecules coordinated to the cage. Hydrogen atoms were included in riding modes. DMA molecules in the void space structure were refined anisotropically. Several parts of the structure were refined as disordered. Each disordered group was refined over two sites and each had appropriate constraints and restraints applied to ensure that normal geometry and normal displacement behaviour was approximated. DMA molecule in the void space containing the atoms O200, O211N200, N211, C200, C201, C202, C203, C211, C212, C213, C214 was modelled in two positions with an occupancy of 0.46 : 0.54. DMA molecule in the void space containing the atoms O300, O311 N300, N311, C300, C301, C302, C303, C311, C312, C313, C314 was modelled in two positions with an occupancy of 0.67 : 0.33. DMA molecule in the void space containing the atoms O400, O411 N400, N411, C400, C401, C402, C403, C411, C412, C413, C414 was modelled in two positions with an occupancy of 0.81 : 0.18. DMA molecule in the void space containing the atoms O500, O511N500, N511, C500, C501, C502, C503, C511, C512, C513, C514 was modelled in two positions with an occupancy of 0.62 : 0.38. DMA molecule in the void space containing the atoms O600, O611N600, N611, C600, C601, C602, C603, C611, C612, C613, C614 was modelled in two positions with an occupancy of 0.75 : 0.25. To refine the final structure, SQUEEZE was used to calculate the remaining electron density accounting a volume of 609 Å^3^ in the unit cell. This volume corresponds to 164 electrons per unit cell (same as per MOP) being approximately three DMA molecules (144 electrons) and two water molecules (20 electrons).

Selected crystallographic and refinement parameters are given in Table S1. Full details of all structures are given in cif format.

Deposition Number(s) 2245850 (for **1‐DMA**), 2245851 (for **2‐DMA**), and 2245852 (for **3‐DMA**) contain(s) the supplementary crystallographic data for this paper. These data are provided free of charge by the joint Cambridge Crystallographic Data Centre and Fachinformationszentrum Karlsruhe Access Structures service.

## Supporting Information

Additional references cited within the Supporting Information.^[30]^

## Conflict of interest

The authors declare no conflict of interest.

1

## Supporting information

As a service to our authors and readers, this journal provides supporting information supplied by the authors. Such materials are peer reviewed and may be re‐organized for online delivery, but are not copy‐edited or typeset. Technical support issues arising from supporting information (other than missing files) should be addressed to the authors.

Supporting Information

## Data Availability

The data that support the findings of this study are openly available from the University of Strathclyde KnowledgeBase at https://doi.org/10.15129/d1c8b4eb‐6dec‐4237‐8cd4‐384717ce0c9f.
