# Supplementary material for: Porous Metal‐Organic Cages Based on Rigid Bicyclo[2.2.2]oct‐7‐ene Type Ligands: Synthesis, Structure, and Gas Uptake Properties
Source: Chemistry. 2023 Apr 25;29(32):e202300732. doi: 10.1002/chem.202300732 (PMC10947411; doi:10.1002/chem.202300732)
Supplement: Supplementary file 1 — Supporting Information [file CHEM-29-0-s001.pdf]

Chemistry–A European Journal

Supporting Information

# Porous Metal-Organic Cages Based on Rigid Bicyclo[2.2.2]oct-7-ene Type Ligands: Synthesis, Structure, and Gas Uptake Properties

Beatriz Doñagueda Suso, Alexandre Legrand, Catherine Weetman, Alan R. Kennedy, Ashleigh J. Fletcher, Shuhei Furukawa, and Gavin A. Craig*

**Table S1.** Selected crystallographic data for compounds **1-DMA**, **2-DMA**, and **3-DMA** 1

**Fig. S1.** $^{1}$H NMR spectrum for **MeOLH$_2$** 2

**Fig. S2.** $^{13}$C NMR spectrum for **MeOLH$_2$** 2

**Fig. S3.** IR spectra for the ligands **MeOLH$_2$**, **CH$_3$LH$_2$**, and **BrLH$_2$** 3

**Fig. S4.** $^{1}$H NMR spectrum for **CH$_3$LH$_2$** 4

**Fig. S5.** $^{13}$C NMR spectrum for **CH$_3$LH$_2$** 4

**Fig. S6.** $^{1}$H NMR spectrum for **BrLH$_2$** 5

**Fig. S7.** $^{13}$C NMR spectrum for **BrLH$_2$** 5

**Fig. S8.** PXRD data for the different phases of **1** 6

**Fig. S9.** PXRD data for the different phases of **2** 7

**Fig. S10.** PXRD data for the different phases of **3** 8

**Fig. S11.** $^{1}$H NMR spectrum for the digestion of **1-DMA** 9

**Fig. S12.** $^{1}$H NMR spectrum for the digestion of **2-DMA** 9

**Fig. S13.** $^{1}$H NMR spectrum for the digestion of **3-DMA** 10

**Fig. S14.** TGA data for **1-DMA** and **1-MeOH** 11

**Fig. S15.** TGA data for **2-DMA** and **2-MeOH** 12

**Fig. S16.** TGA data for **3-DMA** and **3-MeOH** 13

**Fig. S17.** View of the distortion in **1-DMA** 14

**Fig. S18.** View of the distortion in **2-DMA** 15

**Fig. S19.** View of the distortion in **3-DMA** 16

**Fig. S20.** View of the distortion in the cage structure calculated by DFT 17

**Table S2 – S7.** CSD REF codes and data used to make Figure 3 of main manuscript 18 -20

**Fig. S21.** View of the *bc*-plane in **1-DMA** 21

**Fig. S22.** View along the *b*-axis of **1-DMA** 22

**Fig. S23.** View perpendicular to the (1 0 −1) plane in **2-DMA** 23

**Fig. S24.** View of how the coordinated DMA molecule on one MOP slots into the space between MOPs in an adjacent layer in **2-DMA** 24

**Fig. S25.** View parallel to the (1 0 −1) plane in **2-DMA**, showing stacks of MOPs 25

**Fig. S26.** IR spectra for the various phases of **1-DMA** 26

**Fig. S27.** IR spectra for the various phases of **2-DMA** 27

**Fig. S28.** IR spectra for the various phases of **3-DMA** 28

**Fig. S29.** $^{1}H$ NMR spectrum for the digestion of **1-MeOH** 29

**Fig. S30.** $^{1}H$ NMR spectrum for the digestion of **2-MeOH** 29

**Fig. S31.** $^{1}H$ NMR spectrum for the digestion of **3-MeOH** 30

**Fig. S32.** Analysis of surface area of **2a** 31

**Fig. S33.** IR spectrum of **1-post sorption** 32

**Fig. S34.** IR spectrum of **2-post sorption** 33

**Fig. S35.** IR spectrum of **3-post sorption** 34

**Fig. S36.** PXRD data for **1-post sorption** 35

**Fig. S37.** PXRD data for **2-post sorption** 36

**Fig. S38.** PXRD data for **3-post sorption** 37

**Fig. S39.** $^{1}H$ NMR spectrum for the digestion of **1a-post sorption** 38

**Fig. S40.** $^{1}H$ NMR spectrum for the digestion of **2a-post sorption** 38

**Fig. S41.** $^{1}H$ NMR spectrum for the digestion of **3a-post sorption** 39

**Fig. S42.** $^{1}H$ NMR spectrum for the digestion of **1b-post sorption** 39

**Fig. S43.** $^{1}H$ NMR spectrum for the digestion of **2b-post sorption** 40

**Fig. S44.** $^{1}H$ NMR spectrum for the digestion of **3b-post sorption** 40

**Fig. S45.** Analysis of surface area of **2b** 41

**Table S8.** Cartesian coordinates for the model cage calculated by DFT. 42

**Table S1.** Selected crystallographic data for compounds **1-DMA**, **2-DMA**, and **3-DMA**.

| | 1-DMA | 2-DMA | 3-DMA |
|---|---|---|---|
| $\lambda$ (Å) | | 1.54184 | |
| $T$ (K) | | 100(2) | |
| Crystal System | Monoclinic | Monoclinic | Triclinic |
| Space Group | $C2/c$ | $P2_1/n$ | $P$-1 |
| $a$ (Å) | 36.9578(2) | 19.9351(1) | 16.3247(4) |
| $b$ (Å) | 16.9662(1) | 16.6454(1) | 18.2080(5) |
| $c$ (Å) | 30.5900(2) | 25.7952(1) | 19.1451(4) |
| $\alpha$ (°) | 90 | 90 | 63.420(2) |
| $\beta$ (°) | 105.681(1) | 96.853(1) | 67.875(2) |
| $\gamma$ (°) | 90 | 90 | 68.015(2) |
| $V$ (Å$^3$) | 18467.1(2) | 8498.4(1) | 4566.7(2) |
| $Z$ | 4 | 2 | 1 |
| $2\vartheta_{max}$ (°) | 146.44 | 146.40 | 146.60 |
| Reflections | 103433 | 91855 | 93188 |
| Unique Data | 18382 | 16952 | 18235 |
| Observed Data | 16592 | 16075 | 15792 |
| No. Parameters | 1098 | 774 | 1301 |
| $R_{int}$ | 0.0308 | 0.0192 | 0.0319 |
| $R[F^2 > 2\sigma(F^2)]$ | 0.0399 | 0.0490 | 0.0412 |
| $wR(F^2)$ | 0.1143 | 0.1373 | 0.1158 |
| $S$ | 1.039 | 1.117 | 1.062 |
| $\rho_{max}$, $\rho_{min}$ ($e$Å$^{-3}$) | 0.606, −0.796 | 0.881, −0.450 | 0.743, −1.042 |

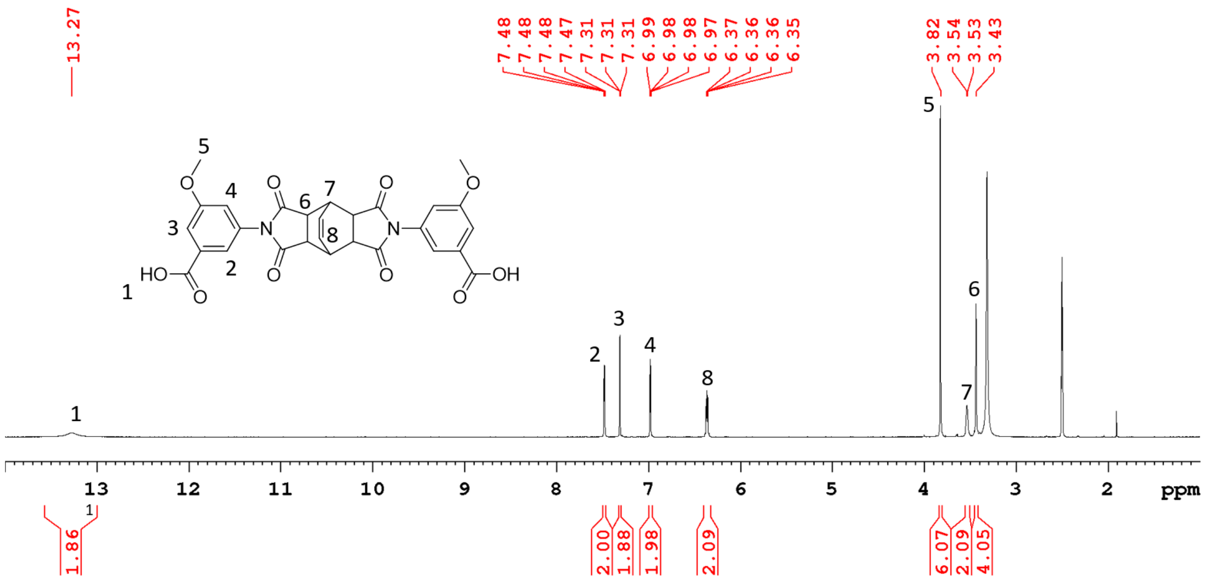


**Figure S1.** $^{1}H$ NMR spectrum for **MeOLH$_2$**. The unassigned peaks at 3.33, 2.50, and 1.95 ppm, correspond to $H_2O$, DMSO-$d_6$, and some residual acetic acid from the synthesis, respectively.

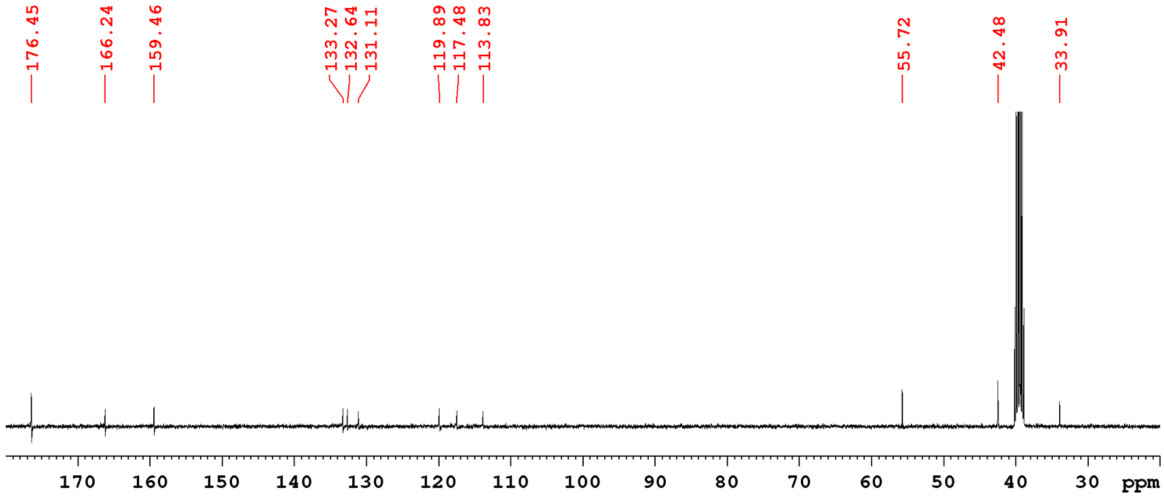


**Figure S2.** $^{13}C$ NMR spectrum for **MeOLH$_2$**.

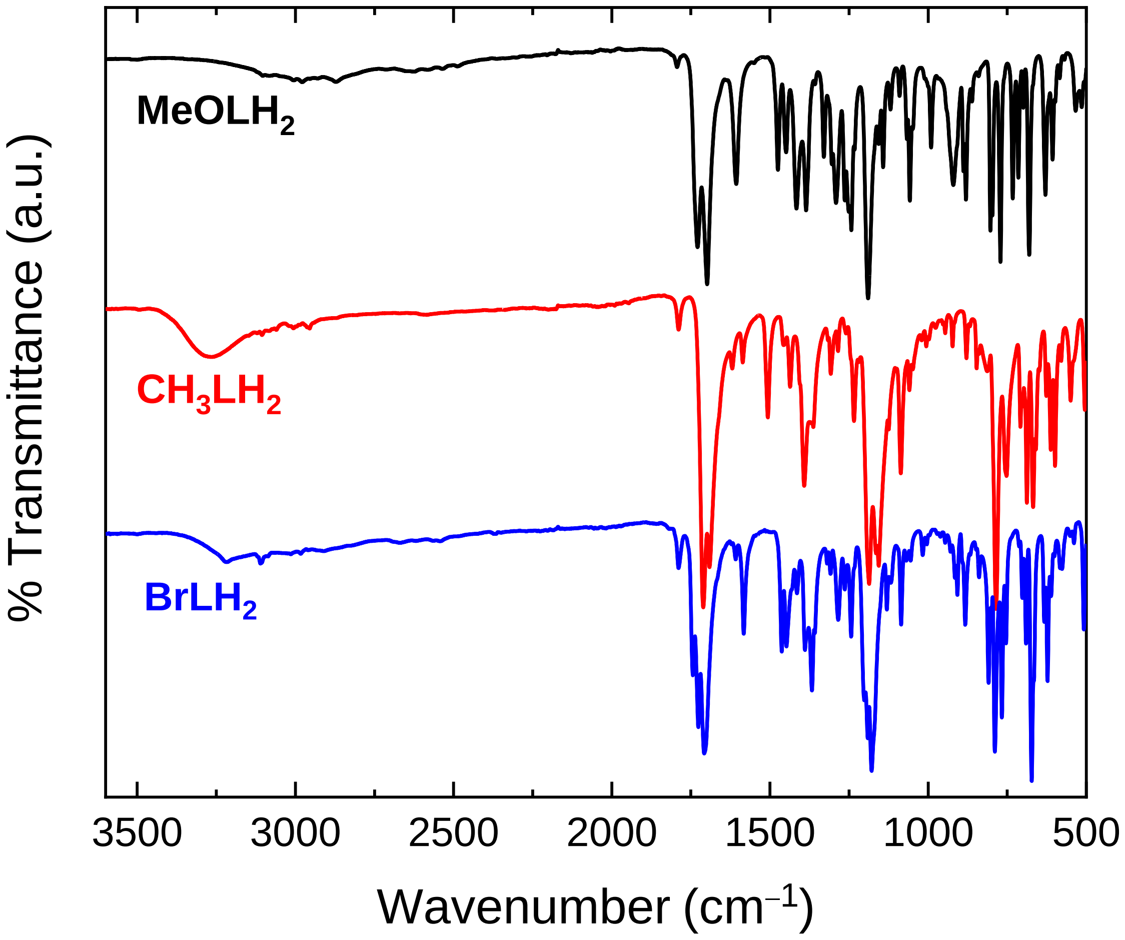


**Figure S3.** Infra-red spectra for the ligands **$MeOLH_2$**, **$CH_3LH_2$**, and **$BrLH_2$**.

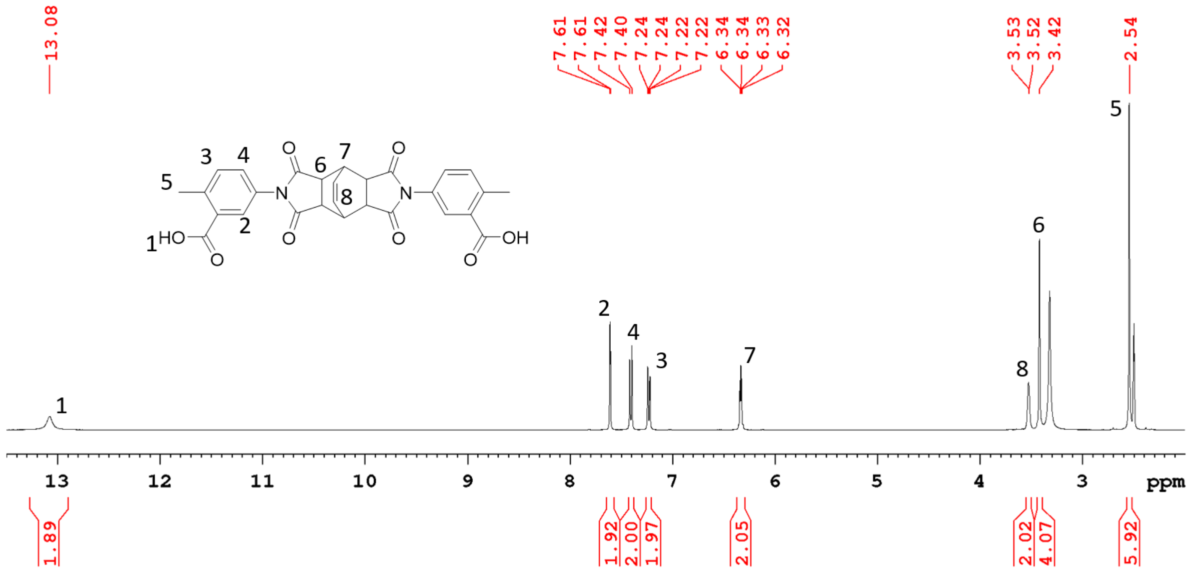


**Figure S4.** ¹H NMR spectrum for **$CH_3LH_2$**. The unassigned peaks at 3.33, and 2.50 ppm, correspond to $H_2O$, and DMSO-$d_6$, respectively.

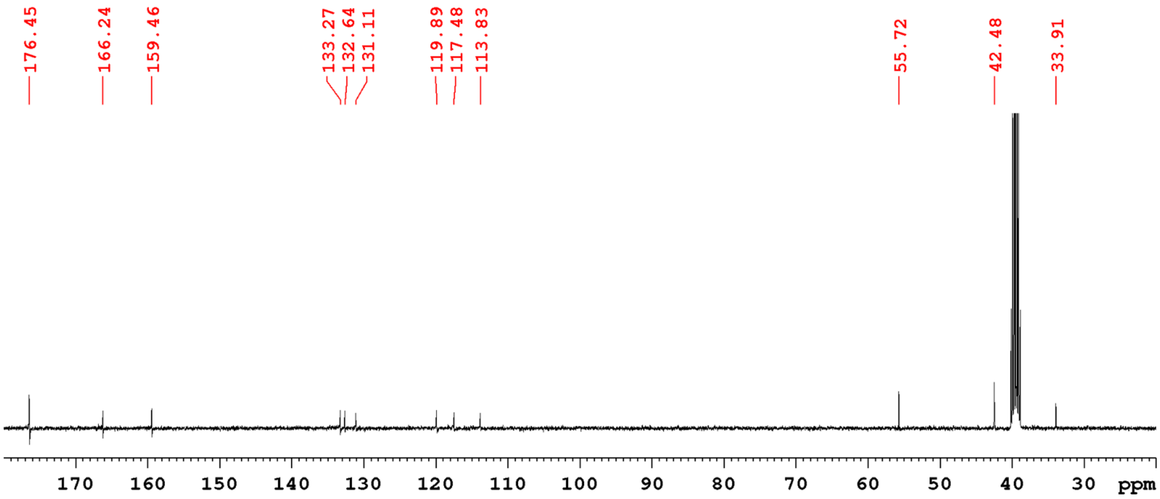


**Figure S5.** ¹³C NMR spectrum for **$CH_3LH_2$**.

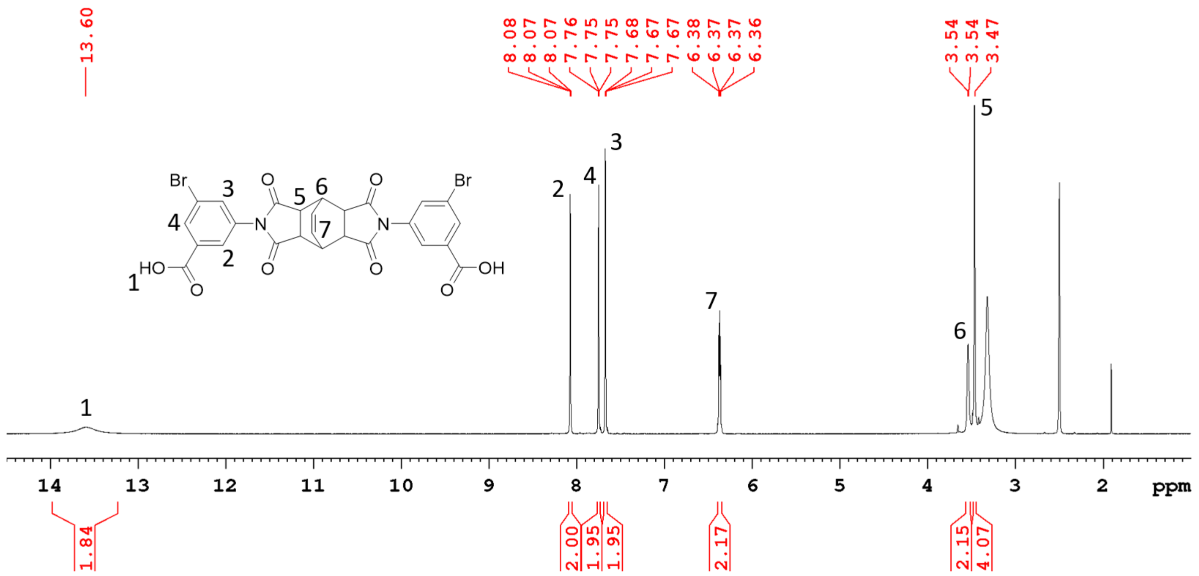


**Figure S6.** $^{1}H$ NMR spectrum for **BrLH₂**. The unassigned peaks at 3.33, 2.50, and 1.95 ppm, correspond to $H_2O$, DMSO-$d_6$, and some residual acetic acid from the synthesis, respectively.

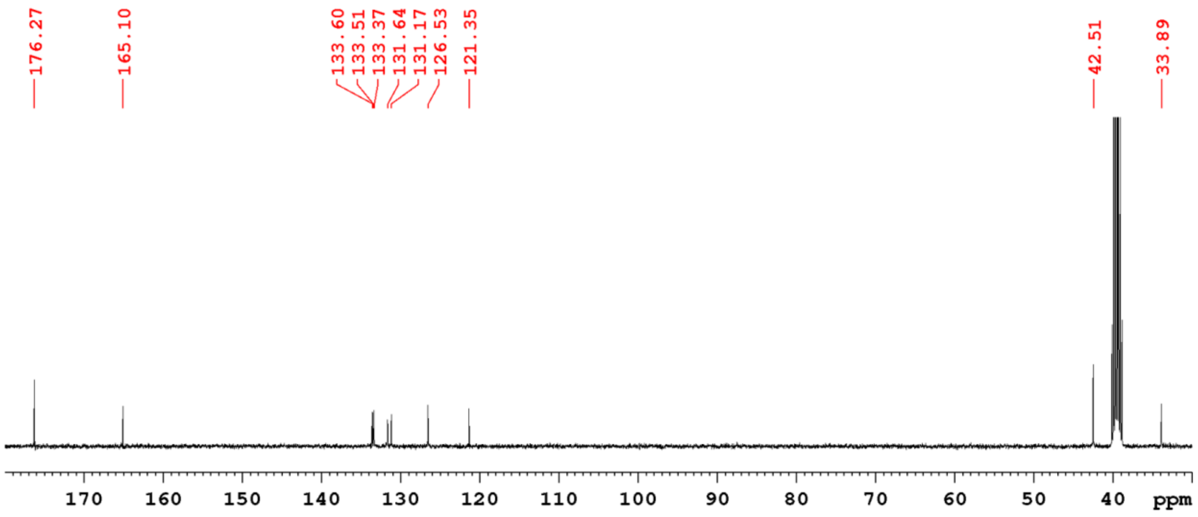


**Figure S7.** $^{13}C$ NMR spectrum for **BrLH₂**.

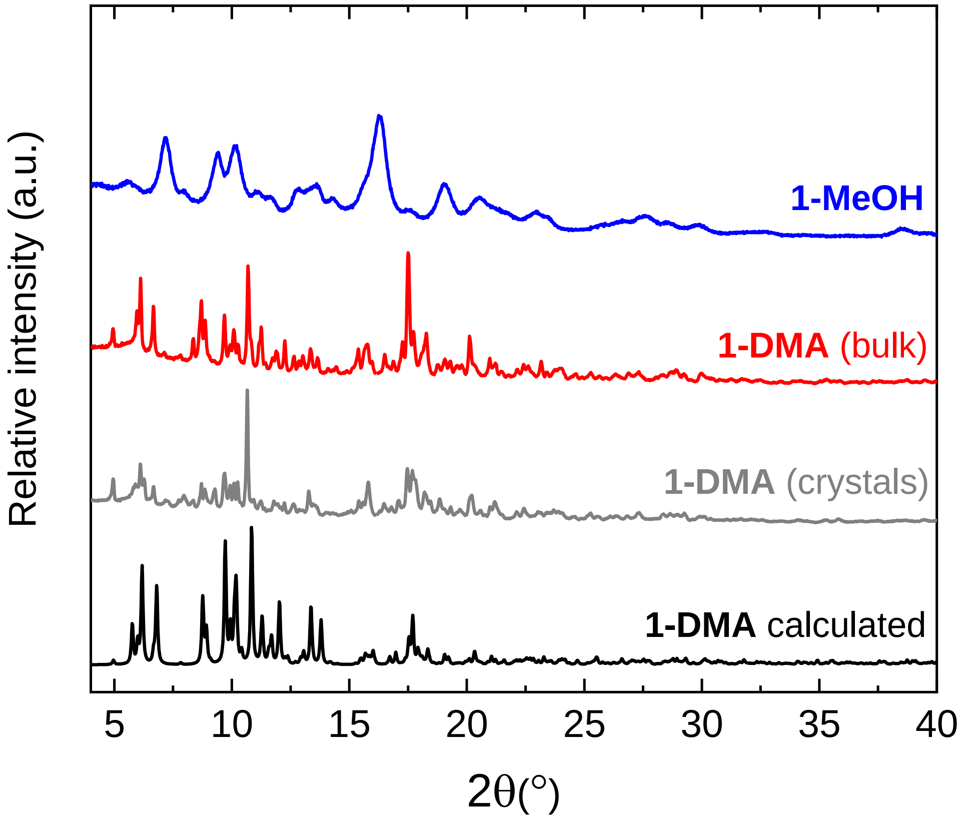


**Figure S8.** Powder X-ray diffractograms for **1-DMA** calculated from .cif data (black), experimental data collected on single crystals (grey), the bulk powder of 1-**DMA** (red), and the phase obtained after MeOH exchange **1-MeOH** (blue).

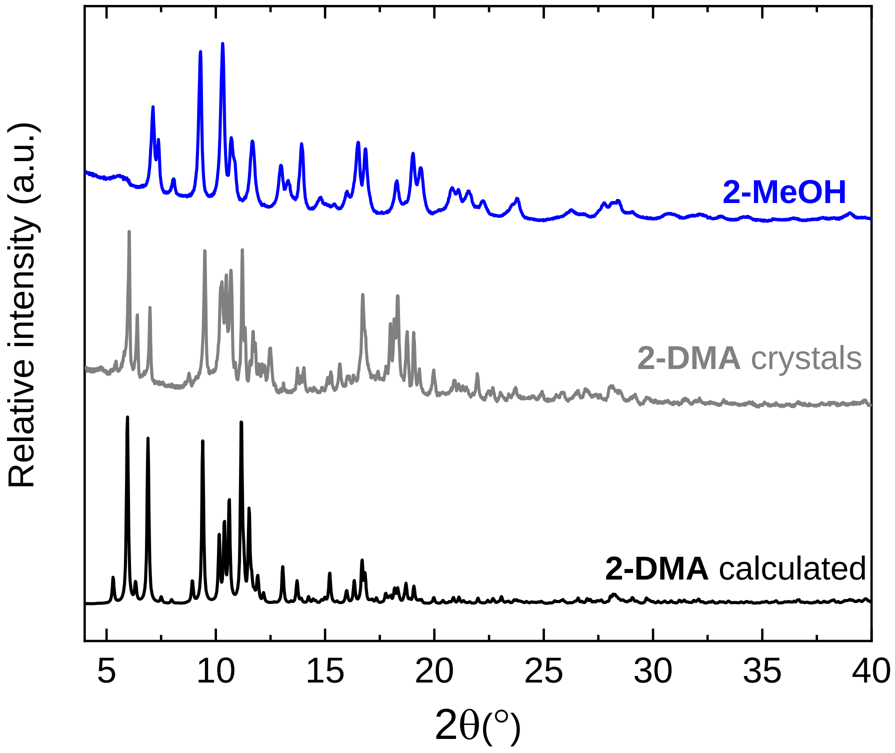


**Figure S9.** Powder X-ray diffractograms for **2-DMA** calculated from .cif data (black), experimental data collected from bulk powder of **2-DMA** (red) and phase obtained after MeOH exchange **2-MeOH** (blue).

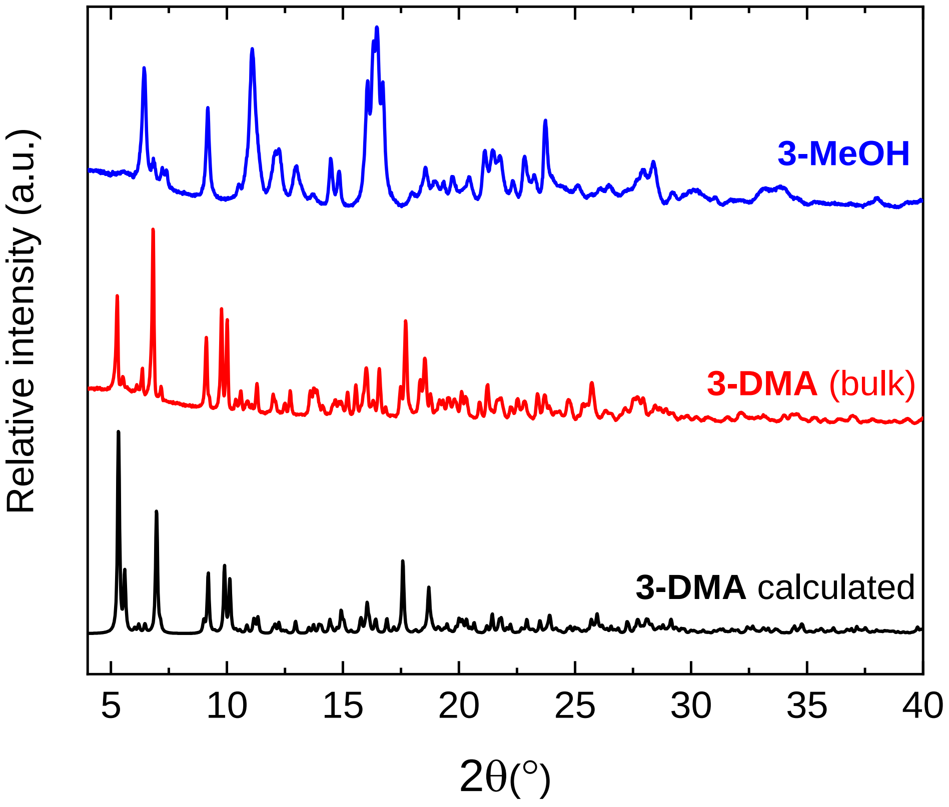


**Figure S10.** Powder X-ray diffractograms for **3-DMA** calculated from .cif data (black), experimental data collected from bulk powder of **3-DMA** (red) and phase obtained after MeOH exchange **3-MeOH** (blue).

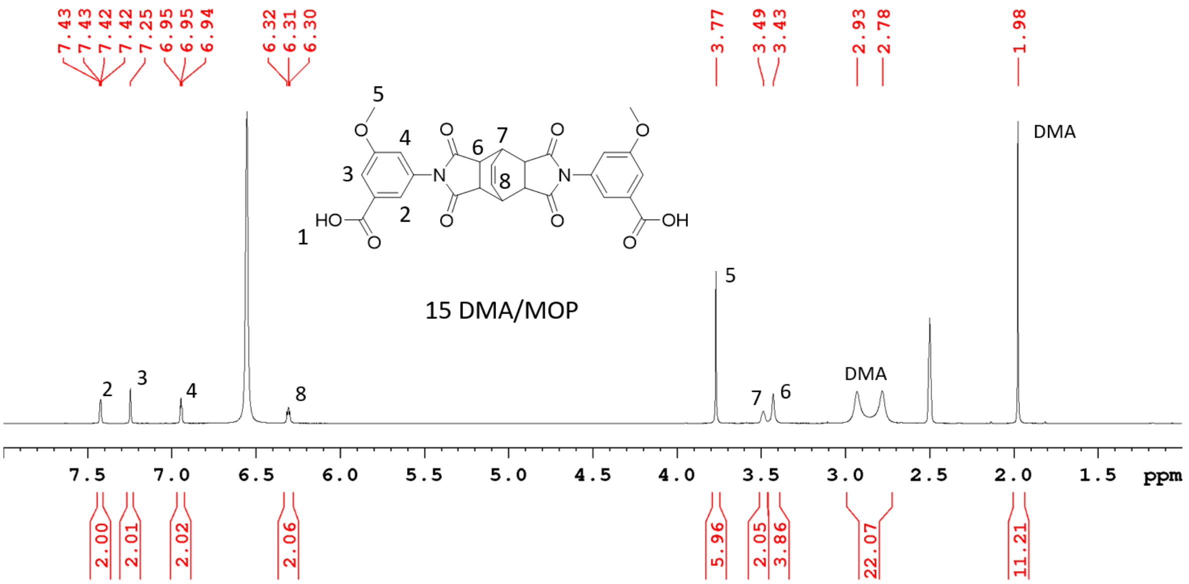


**Figure S11.** $^{1}$H-NMR spectrum of the digestion in DMSO/DCl of **1-DMA** crystals showing the presence of approximately 15 DMA molecules in the crystal structure.

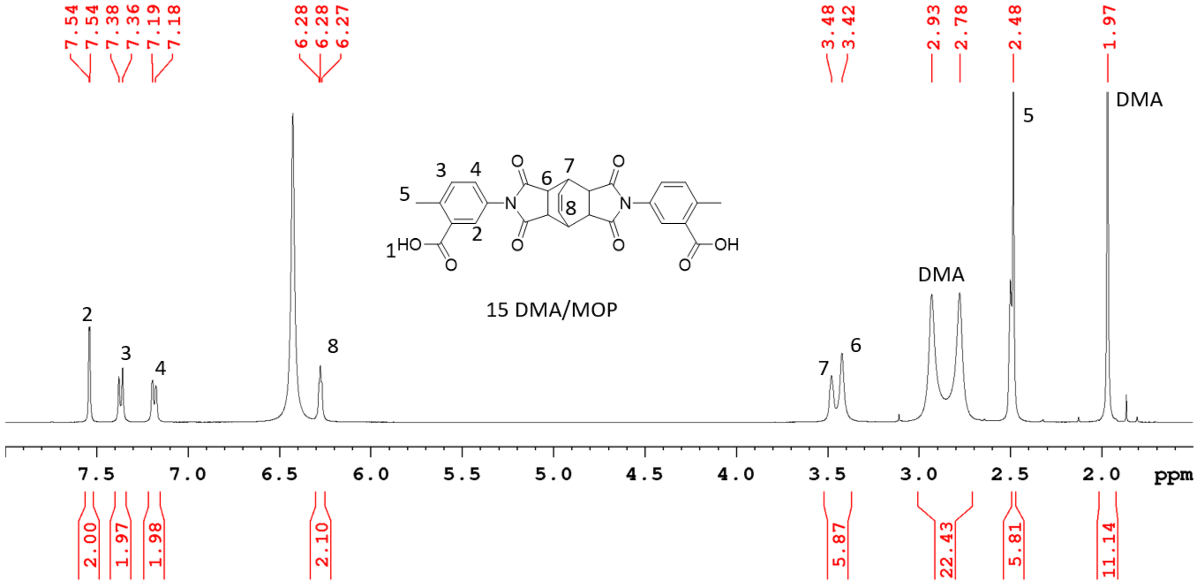


**Figure S12.** $^{1}$H-NMR spectrum of the digestion in DMSO/DCl of **2-DMA** crystals showing the presence of approximately 15 DMA molecules in the crystal structure.

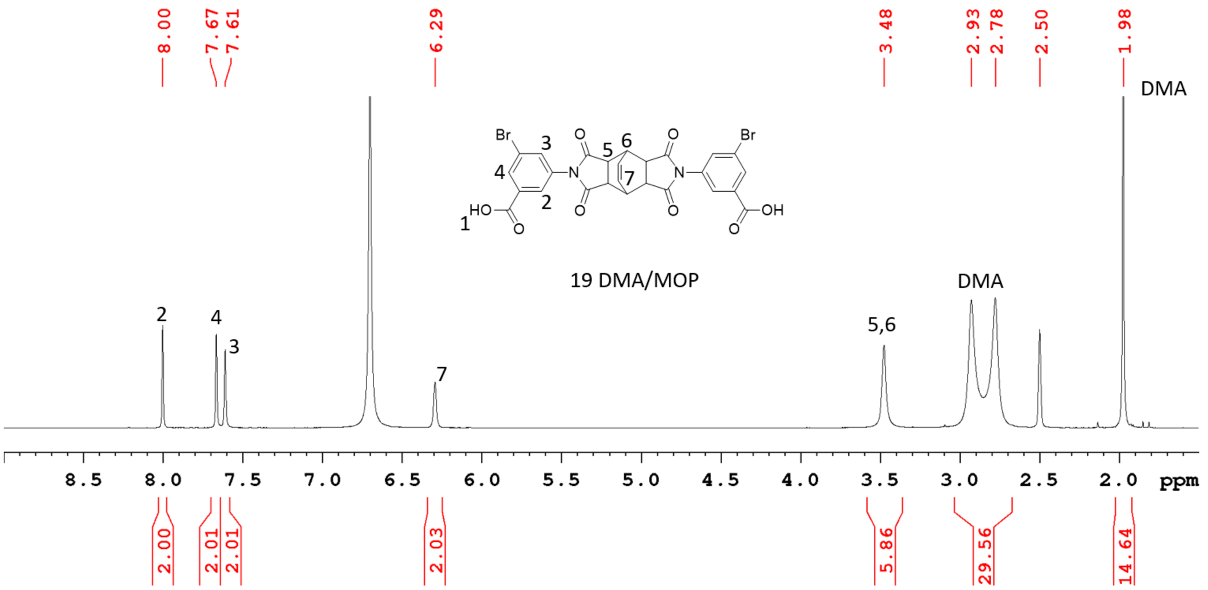


**Figure S13.** $^1$H-NMR spectrum of the digestion in DMSO/DCl of **3-DMA** crystals showing the presence of approximately 19 DMA molecules in the crystal structure.

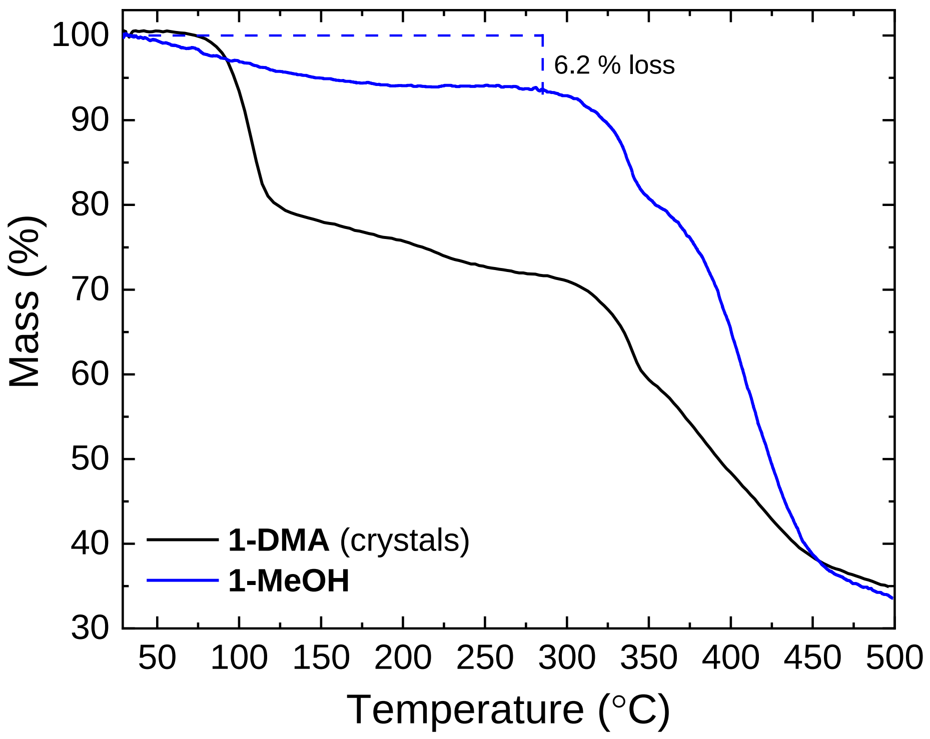


**Figure S14**. TGA trace of crystals of **1-DMA** (black) and bulk sample after solvent exchange with MeOH **1-MeOH** (blue). The mass loss in **1-DMA** is divided into three main steps before the onset of decomposition, which are attributed to the loss of solvent from the pores up to approximately 125 °C, before loss of solvent attached to the paddlewheels and decomposition just above 300 °C. The trace for **1-MeOH** shows a steady loss of solvent before entering a plateau at around 240 °C after a loss of around 6.2 %, which is attributed to the loss of non-coordinated solvent.

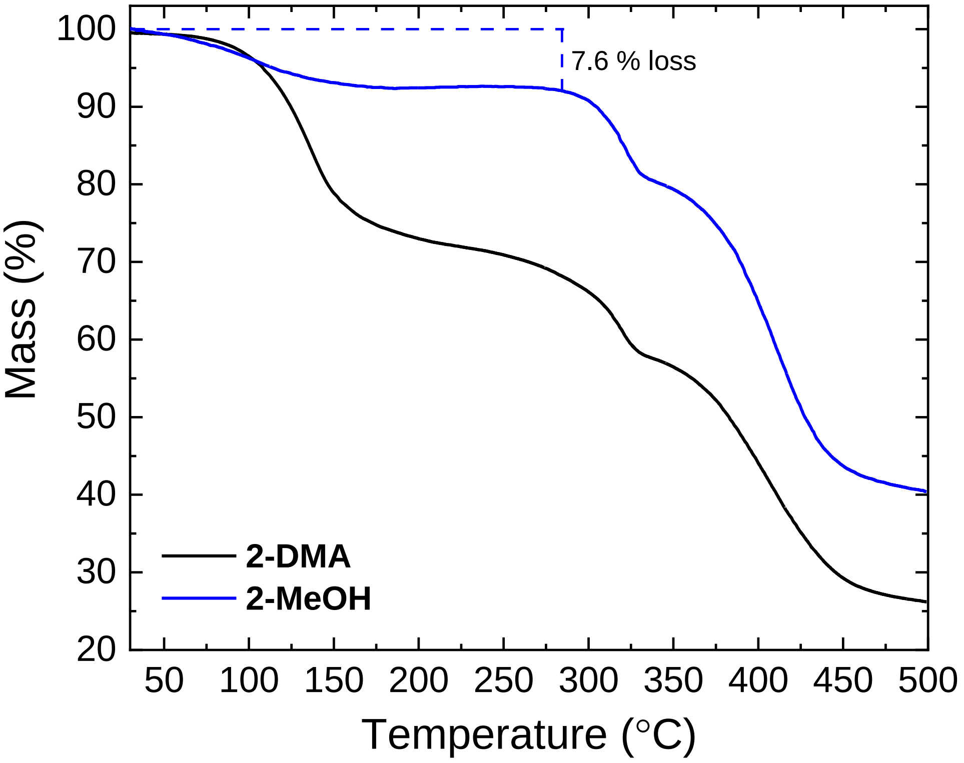


**Figure S15**. TGA trace of crystals of **2-DMA** (black) and bulk sample after solvent exchange with MeOH **2-MeOH** (blue). The mass loss in **2-DMA** is divided into two main steps before the onset of decomposition, which are attributed to the loss of solvent from the pores up to approximately 160 °C, before loss of solvent attached to the paddlewheels and decomposition just above 330 °C. The trace for 2**-MeOH** shows a steady loss of solvent before entering a plateau at around 160 °C after a loss of around 7.6 %, which is attributed to the loss of non-coordinated solvent.

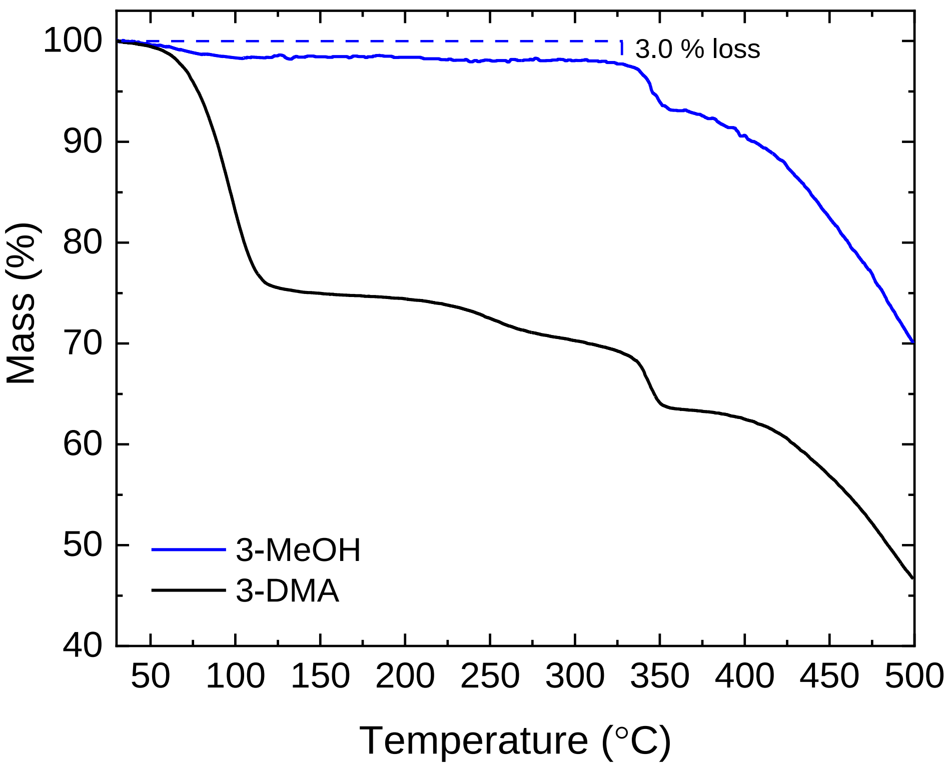


**Figure S16**. TGA trace of crystals of **3-DMA** (black) and bulk sample after solvent exchange with MeOH **3-MeOH** (blue). The mass loss in **3-DMA** is divided into three steps before the onset of decomposition, which are attributed to the loss of solvent from the pores up to approximately 110 °C, before loss of solvent attached to the paddlewheels and decomposition just above 350 °C. The trace for **3-MeOH** shows a steady loss of solvent before entering a plateau at around 160 °C after a loss of around 3.0 %, which is attributed to the loss of non-coordinated solvent.

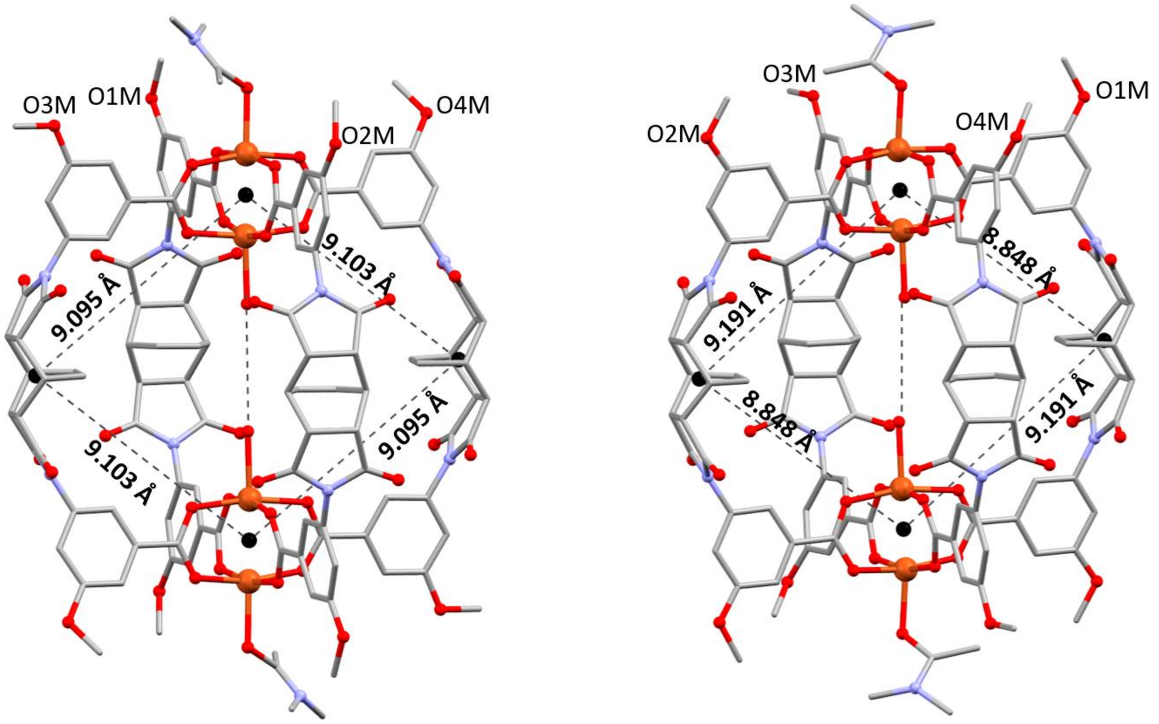


**Figure S17**. The distortion in **1-DMA** as measured by the distance between a centroid defined by the paddlewheel units and a centroid defined by the bicyclooctene units of the coordinating ligands. Connection of these centroids traces out a parallelogram, showing unequal distortion of the cage. Hydrogen atoms have been omitted for clarity, and the ligand substituents are labelled to show the orientation of the cage. Carbon: grey; nitrogen: light purple; oxygen: red; copper: rust.

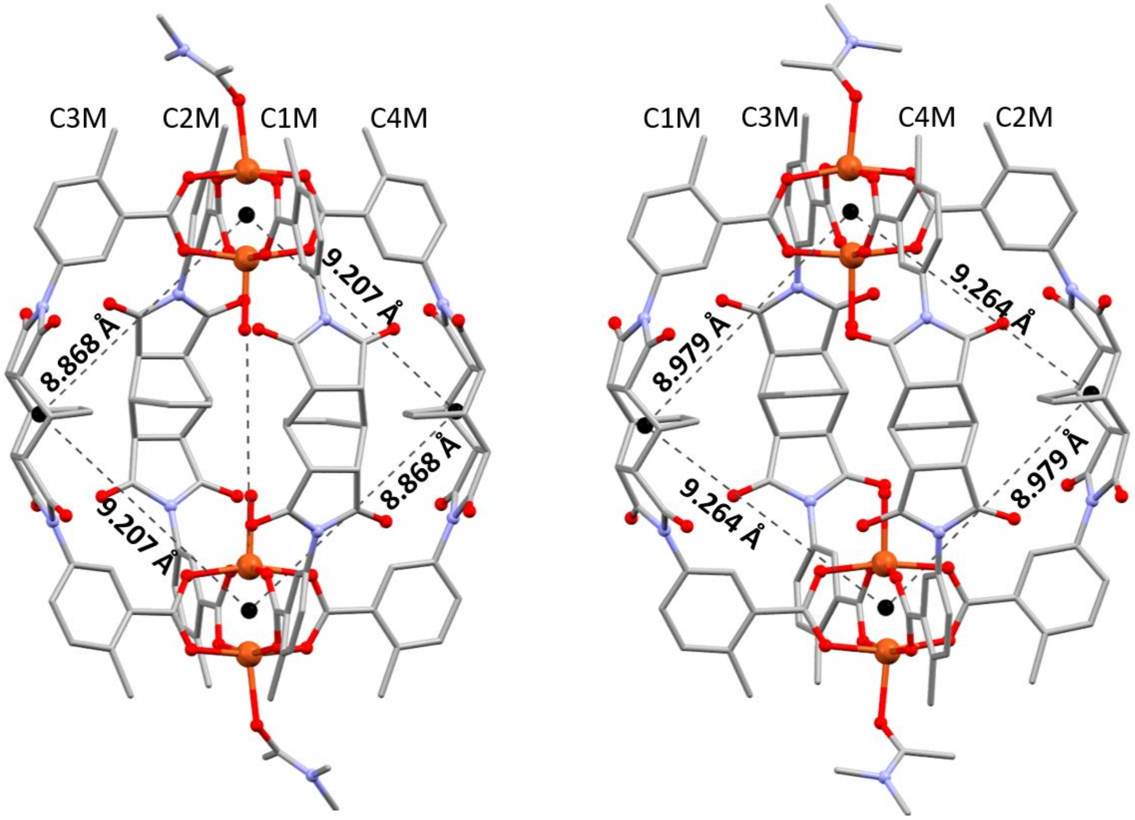


**Figure S18**. The distortion in **2-DMA** as measured by the distance between a centroid defined by the paddlewheel units and a centroid defined by the bicyclooctene units of the coordinating ligands. Connection of these centroids traces out a parallelogram, showing unequal distortion of the cage. Hydrogen atoms have been omitted for clarity, and the ligand substituents are labelled to show the orientation of the cage. Carbon: grey; nitrogen: light purple; oxygen: red; copper: rust.

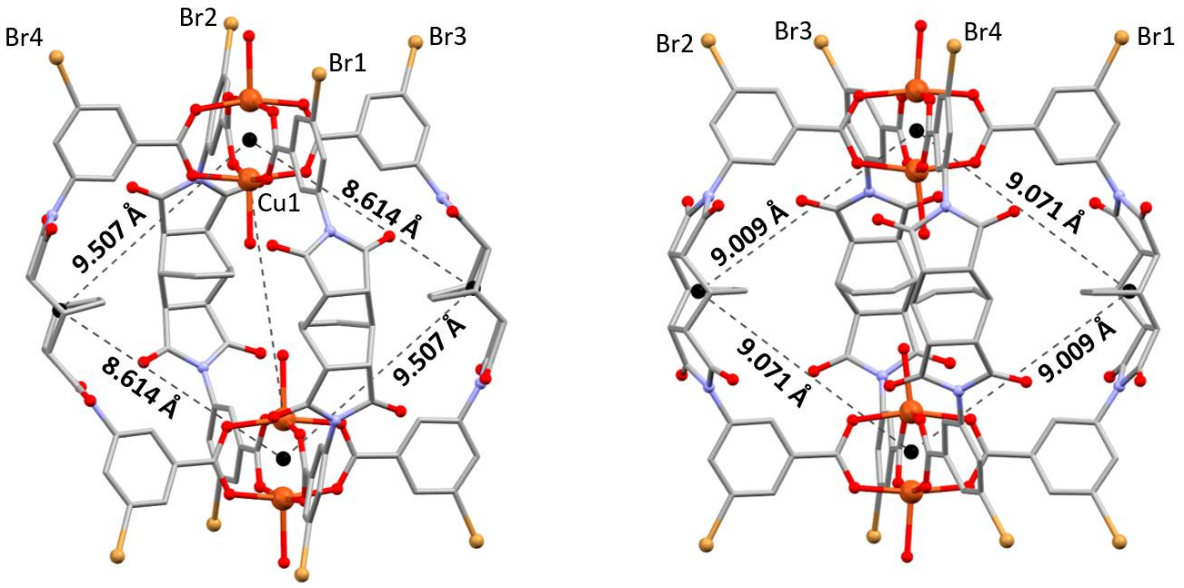


**Figure S19**. The distortion in **3-DMA** as measured by the distance between a centroid defined by the paddlewheel units and a centroid defined by the bicyclooctene units of the coordinating ligands. Connection of these centroids traces out a parallelogram, showing unequal distortion of the cage. Hydrogen atoms have been omitted for clarity, and the ligand substituents are labelled to show the orientation of the cage. Carbon: grey; nitrogen: light purple; oxygen: red; bromine: light orange; copper: rust.

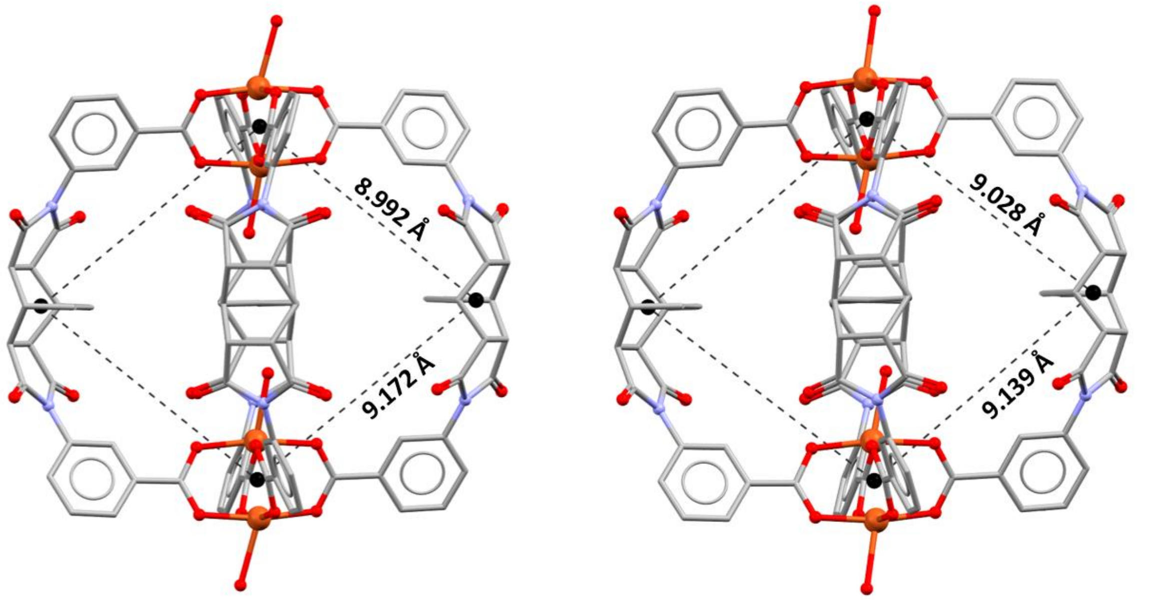


**Figure S20**. The distortion in the cage structure calculated using DFT. Hydrogen atoms have been omitted for clarity, and the ligand substituents are labelled to show the orientation of the cage. Carbon: grey; nitrogen: light purple; oxygen: red; copper: rust.

**Table S2.** The data used to plot Figure 3 of the main manuscript. Where the paddlewheel is not Cu-Cu, the composition is specified.

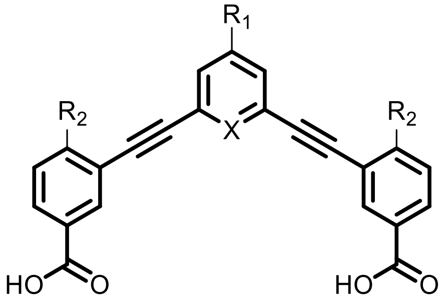


| CSD Ref code | X | R$_1$ | R$_2$ | M···M distance (Å) | Reference (main manuscript) |
|---|---|---|---|---|---|
| EBIKIN | C-NH$_2$ | $^{i}$Pr | H | 9.393 | 30 (a) |
| EQEVOM | C-H | $CH_2OCH_2OH$ | H | 9.240 | 8(d) |
| EQEVIG | C-H | $CH_2OH$ | H | 9.516 | 8(d) |
| EQEVUS | C-H | $CH_2OC(O)C_5H_{11}$ | H | 9.252 | 8(d) |
| EQEWAZ | C-H | $CH_2OC(O)C_{11}H_{23}$ | H | 9.141 | 8(d) |
| GASTAZ | C-H | C(O)H | H | 9.340 (Rh-Rh) | 10(a) |
| IGEBII | C-H | $OC_3H_7$ | H | 8.984 | 8(b) |
| IGEBOO | C-H | $OCH_3$ | H | 9.321 | 8(b) |
| JUZTOQ | C-NH$_2$ | NHC(O)O$^{t}$Bu | H | 9.071 | 8(a) |
| JUZTOQ | C-NH$_2$ | NHC(O)O$^{t}$Bu | H | 8.921 | 8(a) |
| JUZTUW | C-NH$_2$ | NHC(O)OFMOC | H | 9.254 | 8(a) |
| NUSDOV | C-H | H | H | 9.046 | 8(e) |
| NUSDUB | C-H | H | H | 9.405 | 8(e) |
| NUSFAJ | C-H | H | $OCH_3$ | 9.489 | 8(e) |
| PIKDEV | C-H | $OC_2H_5$ | H | 9.308 | 9 |
| PIKDIZ | C-H | $OC_2H_5$ | H | 9.172 | 9 |
| PIKDOF | C-H | $OC_2H_5$ | H | 9.119 (Rh-Rh) | 9 |
| PIKDOF | C-H | $OC_2H_5$ | H | 9.286 (Rh-Rh) | 9 |
| TUDLEM | C-H | $CHNC_6H_4CH_3$ | H | 8.652 | 18 |
| TUDLIQ | C-H | C(O)H | H | 9.416 | 18 |
| TUDLUC | C-H | CHN(9-ethyl-9*H*-carbzol-3-amine) | H | 9.304 | 18 |
| TUDMAJ | C-H | C(O)H | H | 9.464 | 18 |
| TUDMAJ | C-H | C(O)H | H | 9.015 | 18 |
| TUDMEN | C-H | C(O)H | H | 9.101 | 18 |
| TUDMIR | C-H | C(O)H | H | 9.359 | 18 |
| TUDLOW | C-H | C(O)H | H | 9.383 | 18 |
| TUDLOW | C-H | C(O)H | H | 9.018 | 18 |
| XAJDET | C-NH$_2$ | $^{i}$Pr | H | 9.150 | 3(h) |
| XUQJIE | C-H | H | H | 9.668 (Mo-Mo) | 8(f) |
| XUQJOK | C-NH$_2$ | $^{i}$Pr | H | 9.738 (Mo-Mo) | 8(f) |
| XUVHAZ | C-H | H | H | 9.498 (Rh-Rh) | 8(c) |
| YUCFEJ | C-NH$_2$ | $OC_9H_{18}CH_3$ | H | 9.096 | 10(b) |
| YUCFIN | C-NH$_2$ | $OCH_3$ | H | 9.121 | 10(b) |
| YUCFOT | C-NH$_2$ | $OCH_3$ | H | 9.405 | 10(b) |
| 2072629 | C-F | $NH_2$ | H | 9.213 | 30(b) |

**Table S3.** The data used to plot Figure 3 of the main manuscript. Where the paddlewheel is not Cu-Cu, the composition is specified.

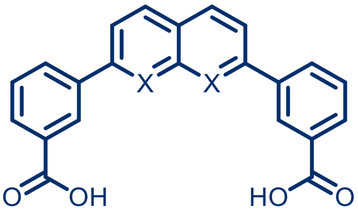


| CSD Ref code | X | M···M distance (Å) | Reference (main manuscript) |
|---|---|---|---|
| NIGFAM | C | 7.431 | 30(c) |
| NIGDUE | N | 6.707 | 30(c) |
| NIGDOY | N | 7.274 (Mo-Mo) | 30(c) |

**Table S4.** The data used to plot Figure 3 of the main manuscript. Where the paddlewheel is not Cu-Cu, the composition is specified.

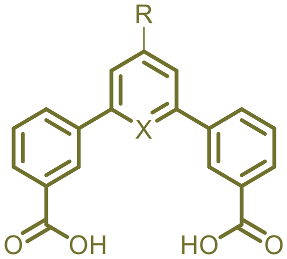


| CSD Ref code | X | R | M···M distance (Å) | Reference (main manuscript) |
|---|---|---|---|---|
| SUVSIO | N | H | 4.497 | 15 |
| SUVVIR | C | $CH_3$ | 5.035 | 15 |
| SUVSEK | C | $CH_3$ | 5.477 (Mo-Mo) | 15 |
| SUVSEK | C | $CH_3$ | 5.663 (Mo-Mo) | 15 |
| SUVSOU | C | $CH_3$ | 5.220 (Cr-Cr) | 15 |
| XUQJEA | N | H | 4.839 (Mo-Mo) | 8(f) |

**Table S5.** The data used to plot Figure 3 of the main manuscript.

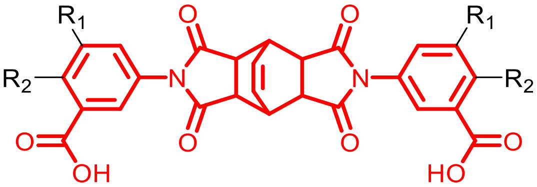


| CSD Ref code | $R_1$ | $R_2$ | M···M distance (Å) | Reference (main manuscript) |
|---|---|---|---|---|
| UTACUO | H | H | 8.566 | 24 |
| | $OCH_3$ | H | | This work |
| | Br | H | | This work |
| | H | $CH_3$ | | This work |

**Table S6.** The data used to plot Figure 3 of the main manuscript. The conformation of the ligands in the cage is indicated.

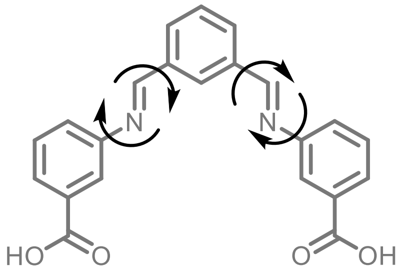


| CSD Ref code | Conformation | M···M distance (Å) | Reference (main manuscript) |
|---|---|---|---|
| ANAKUY | *syn-anti* (all four ligands) | 8.414 | 11 |
| ANALAF | *syn-anti* (two ligands)<br>*anti-anti* (two ligands) | 8.160 | 11 |
| ANALEJ | *syn-anti* (two ligands)<br>*syn-syn* (two ligands) | 8.768 | 11 |
| ANALOT | *syn-anti* (two ligands)<br>*anti-anti* (two ligands) | 8.234 | 11 |
| ANALUZ | *syn-anti* (all four ligands) | 8.421 | 11 |

**Table S7.** The data used to plot Figure 3 of the main manuscript.

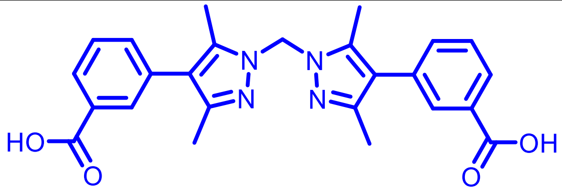


| CSD Ref code | M···M distance (Å) | Reference (main manuscript) |
|---|---|---|
| PEHMAU | 8.414 | 12 |
| PEHMEY | 8.160 | 12 |
| PEHMIC | 8.768 | 12 |
| PEHMOI | 8.234 | 12 |
| PEHMUO | 8.421 | 12 |

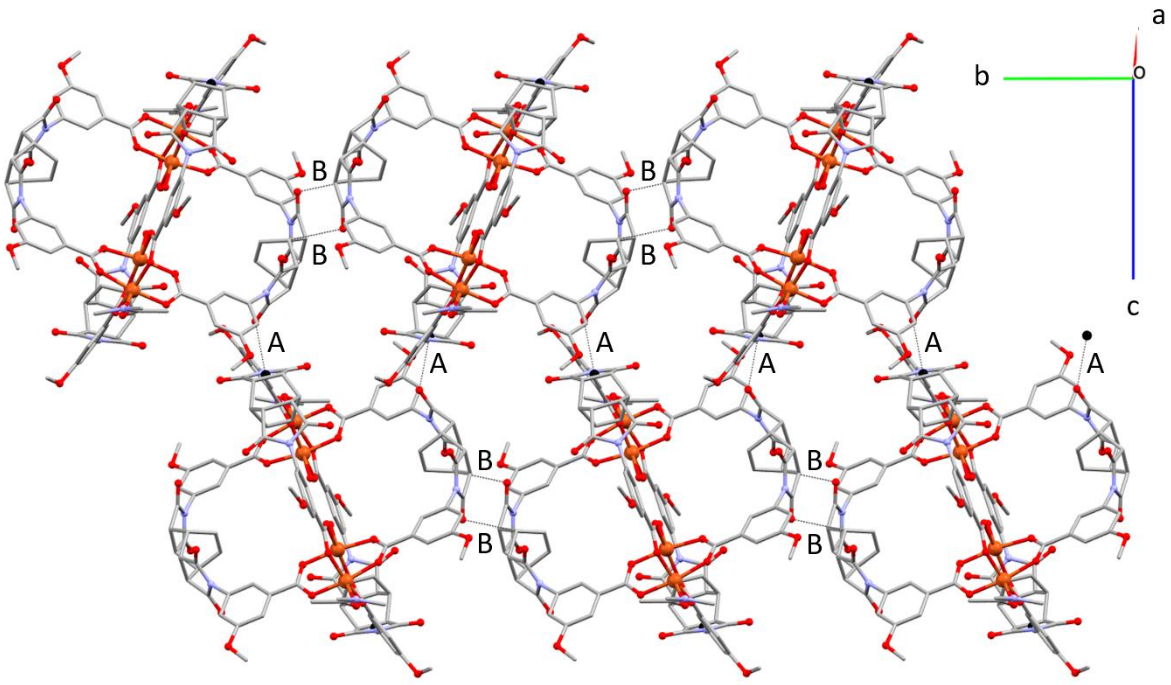


**Figure S21.** View of the *bc*-plane in **1-DMA**. Non-coordinated solvent molecules and hydrogen atoms have been omitted for clarity. The interaction denoted by A corresponds to the distance between O3 and the centroid of a dicarboximide ring, and measures 2.813(2) Å. The interaction denoted by B corresponds to the short contact O2 and C3 of the bicyclooctene ring of a neighbouring cage, and measures 3.172(3) Å. Colour code as in Figure S17.

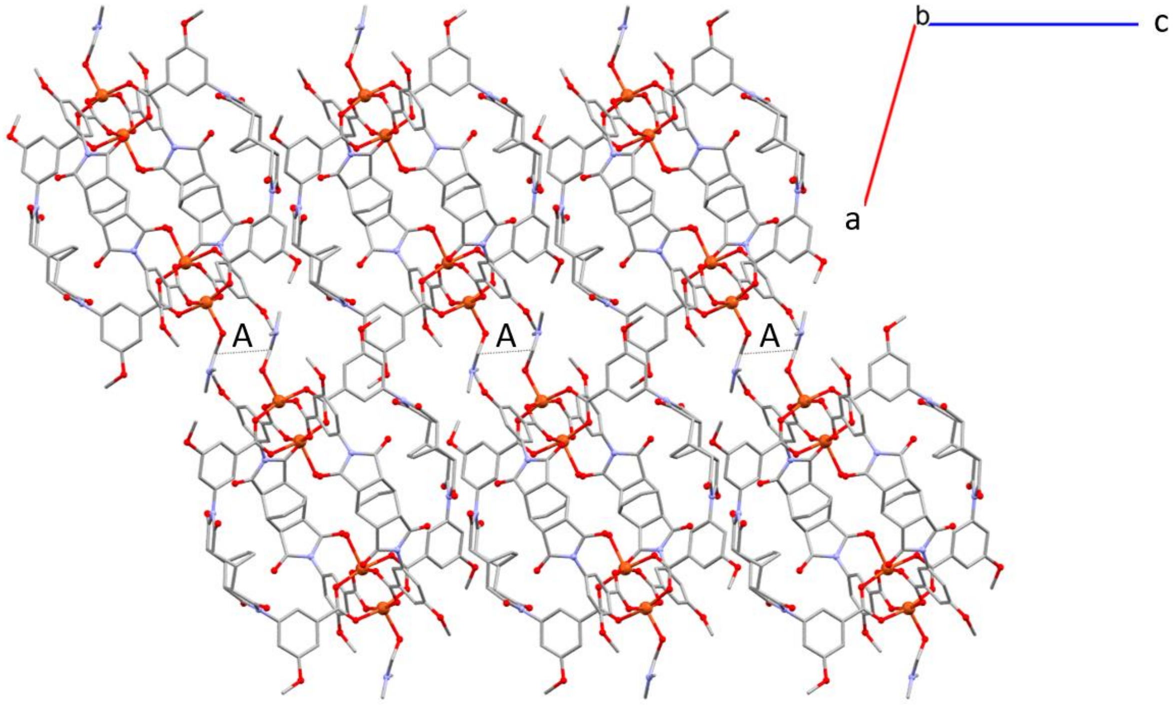


**Figure S22.** View along the *b*-axis of the crystal structure of **1-DMA**, showing the interaction between the coordinated DMA molecules. Non-coordinated solvent molecules and hydrogen atoms have been omitted for clarity. The interaction denoted by A corresponds to the distance symmetry-related C1Z atoms of the coordinated DMA solvent molecules, and measures 3.226(9) Å. Colour code as in Figure S17.

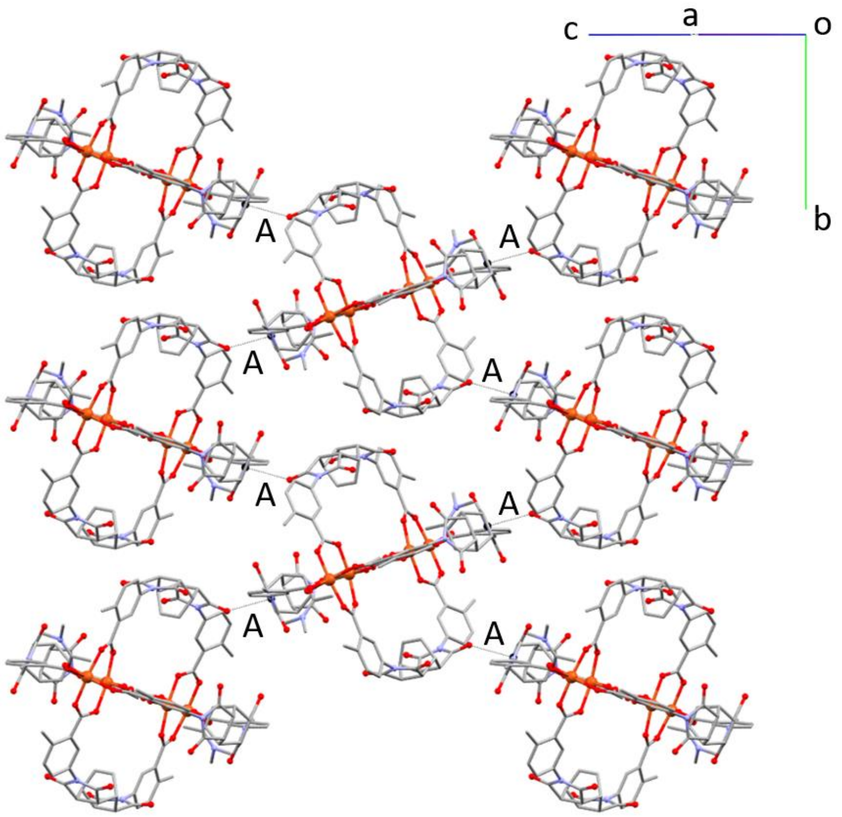


**Figure S23.** View perpendicular to the (1 0 −1) plane in **2-DMA**, showing the interaction between O12 of the dicarboximide ring and the centroid of a dicarboximide ring on a neighbouring MOP, measuring 2.918(2) Å. Each MOP displays four of these interactions within the plane, two as acceptor and two as donor. Non-coordinated solvent molecules and hydrogen atoms have been omitted for clarity. Colour code as in Figure S18.

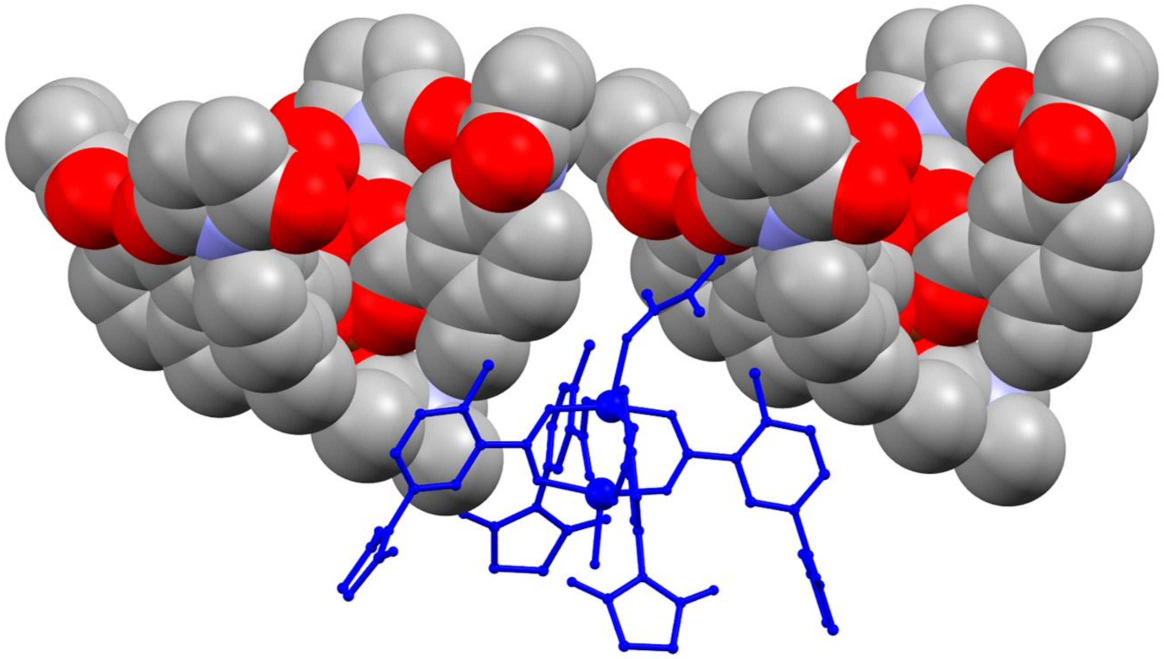

**Figure S24.** View of how the coordinated DMA molecule on one MOP slots into the space between MOPs in an adjacent layer in **2-DMA**. The MOPs in the adjacent layer are shown in spacefill view, with only the bottom half of the MOP shown, while the MOP from the layer below is shown in ball and stick and coloured blue.

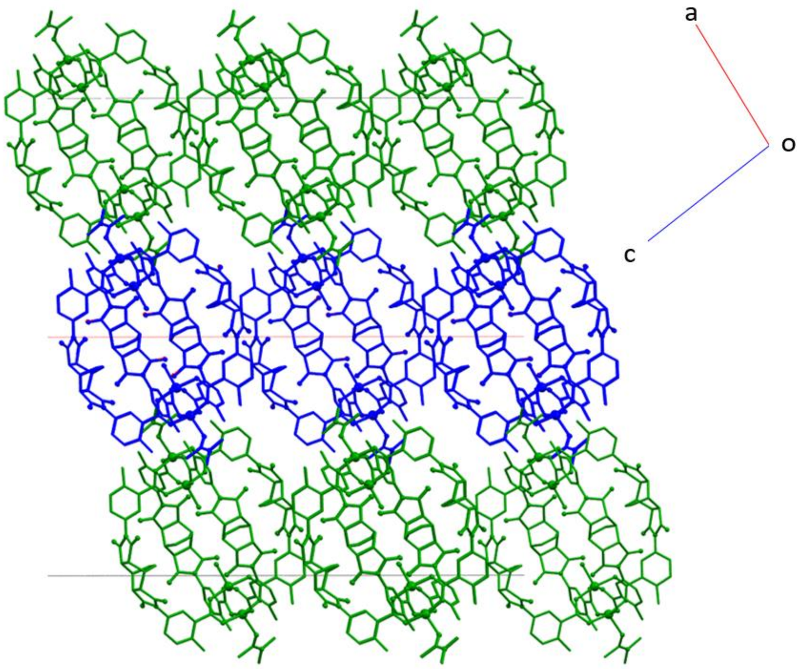


**Figure S25.** View parallel to the (1 0 −1) plane in **2-DMA**, showing alternating layers of MOPs, which interact mostly through dispersion forces. Non-coordinated solvent molecules and hydrogen atoms have been omitted for clarity.

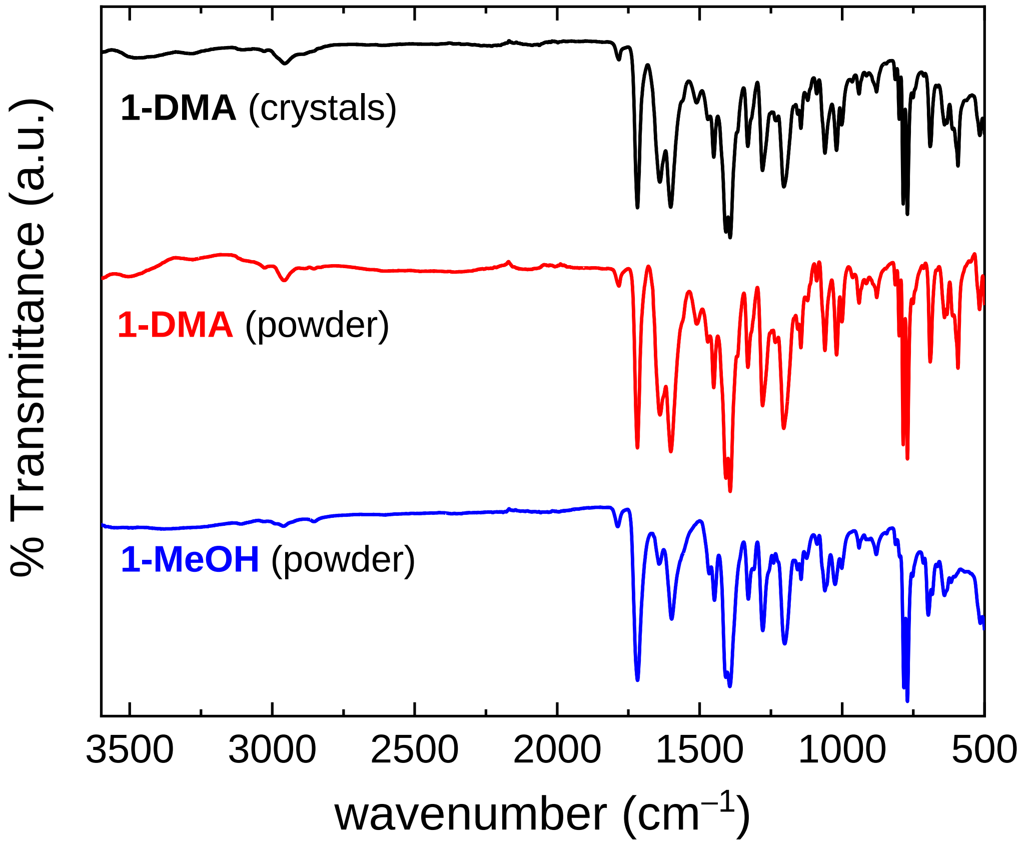


**Figure S26.** IR spectra for the as-synthesised crystals of **1-DMA** (black), the scaled-up bulk powder (red), and the phase obtained after washing with MeOH, **1-MeOH**.

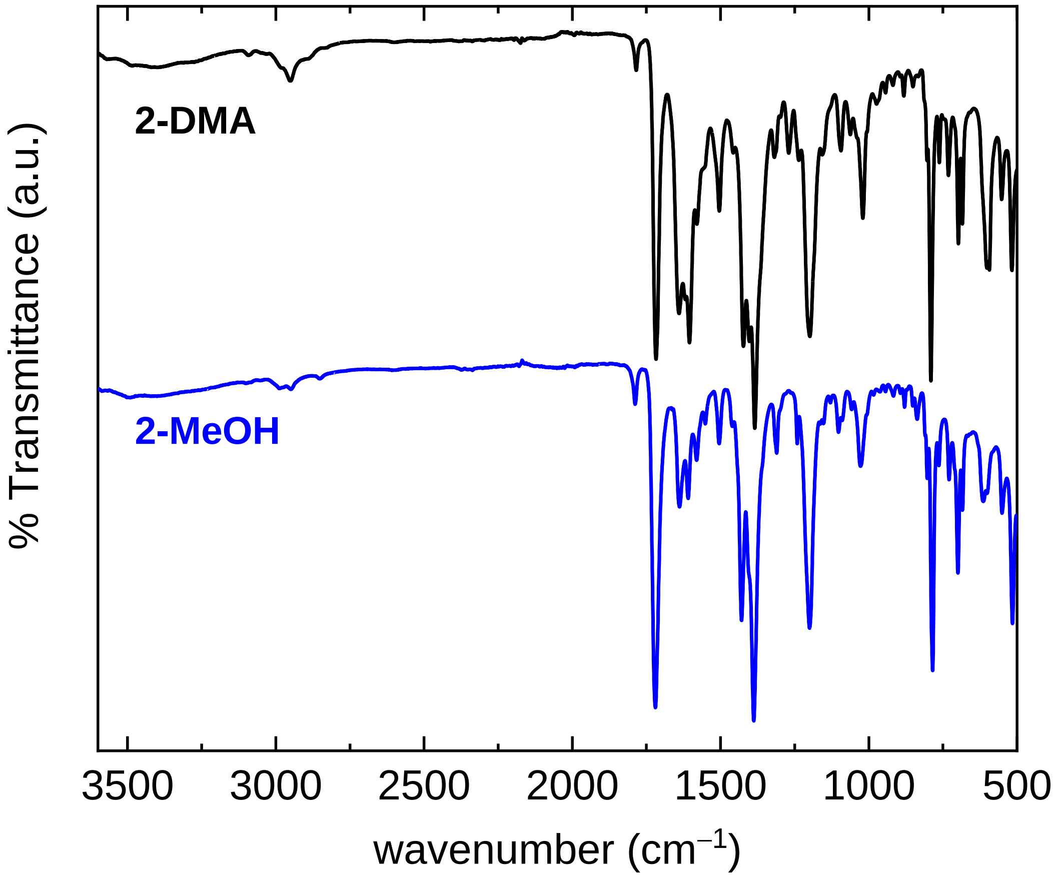


**Figure S27.** IR spectra for the as-synthesised crystals of **2-DMA** (black), and the phase obtained after washing with MeOH, **2-MeOH**.

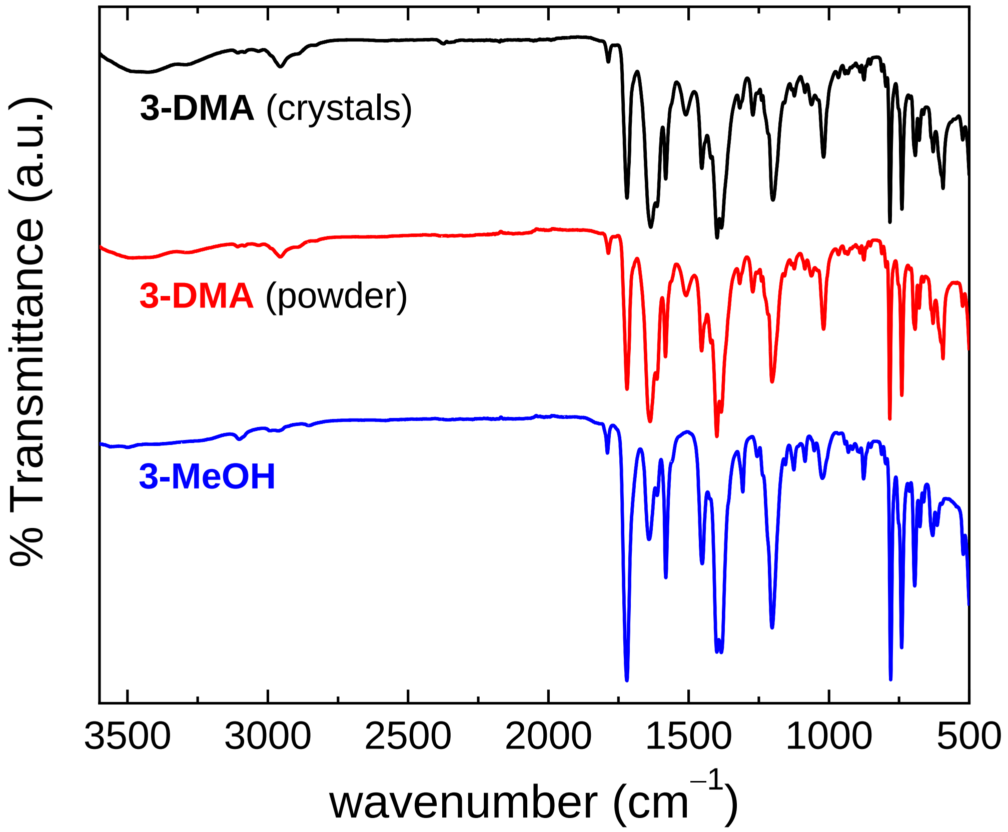


**Figure S28.** IR spectra for the as-synthesised crystals of **3-DMA** (black), the scaled-up bulk powder (red), and the phase obtained after washing with MeOH, **3-MeOH**.

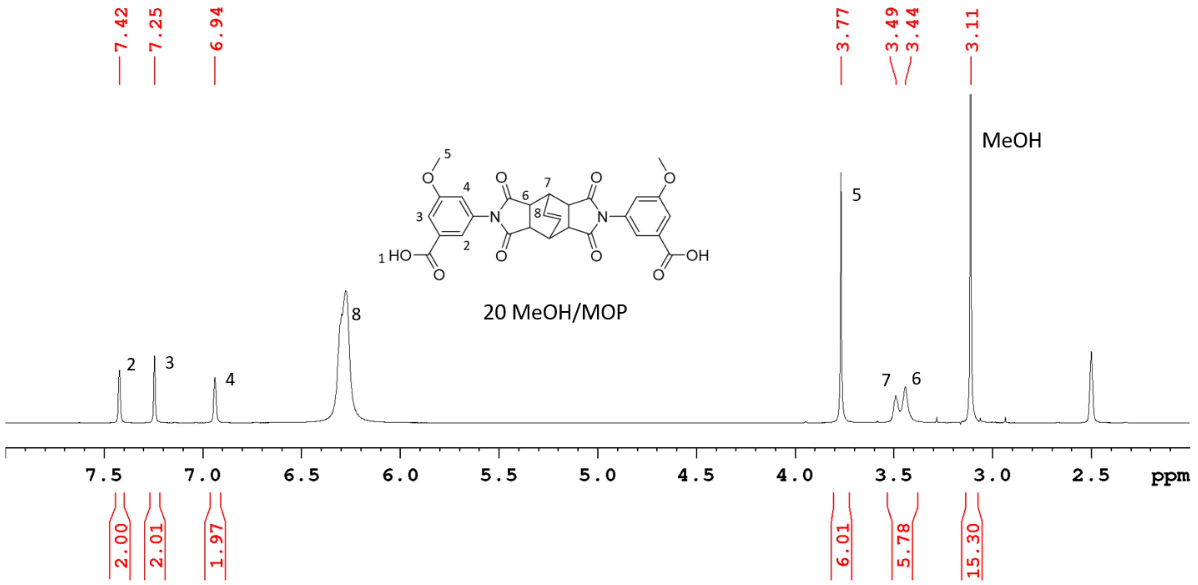


**Figure S29.** $^{1}$H-NMR spectrum of the digestion in DMSO/DCl of **1-MeOH** crystals showing the presence of approximately 20 MeOH molecules in the structure.

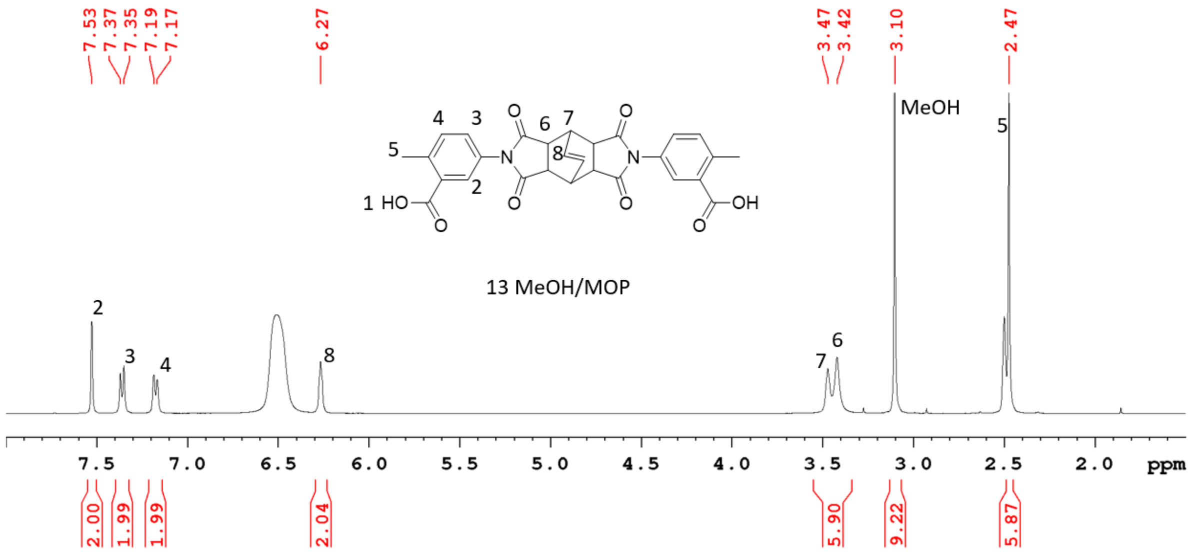


**Figure S30.** $^{1}$H-NMR spectrum of the digestion in DMSO/DCl of **2-MeOH** crystals showing the presence of approximately 13 MeOH molecules in the structure.

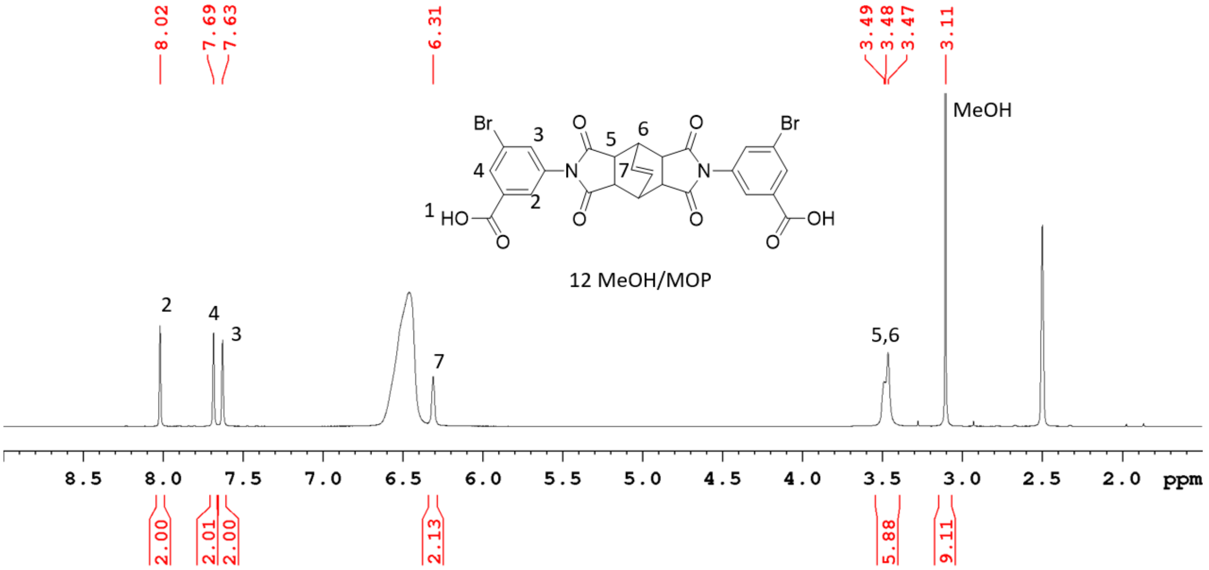


**Figure S31.** $^{1}$H-NMR spectrum of the digestion in DMSO/DCl of **3-MeOH** crystals showing the presence of approximately 12 MeOH molecules in the structure.

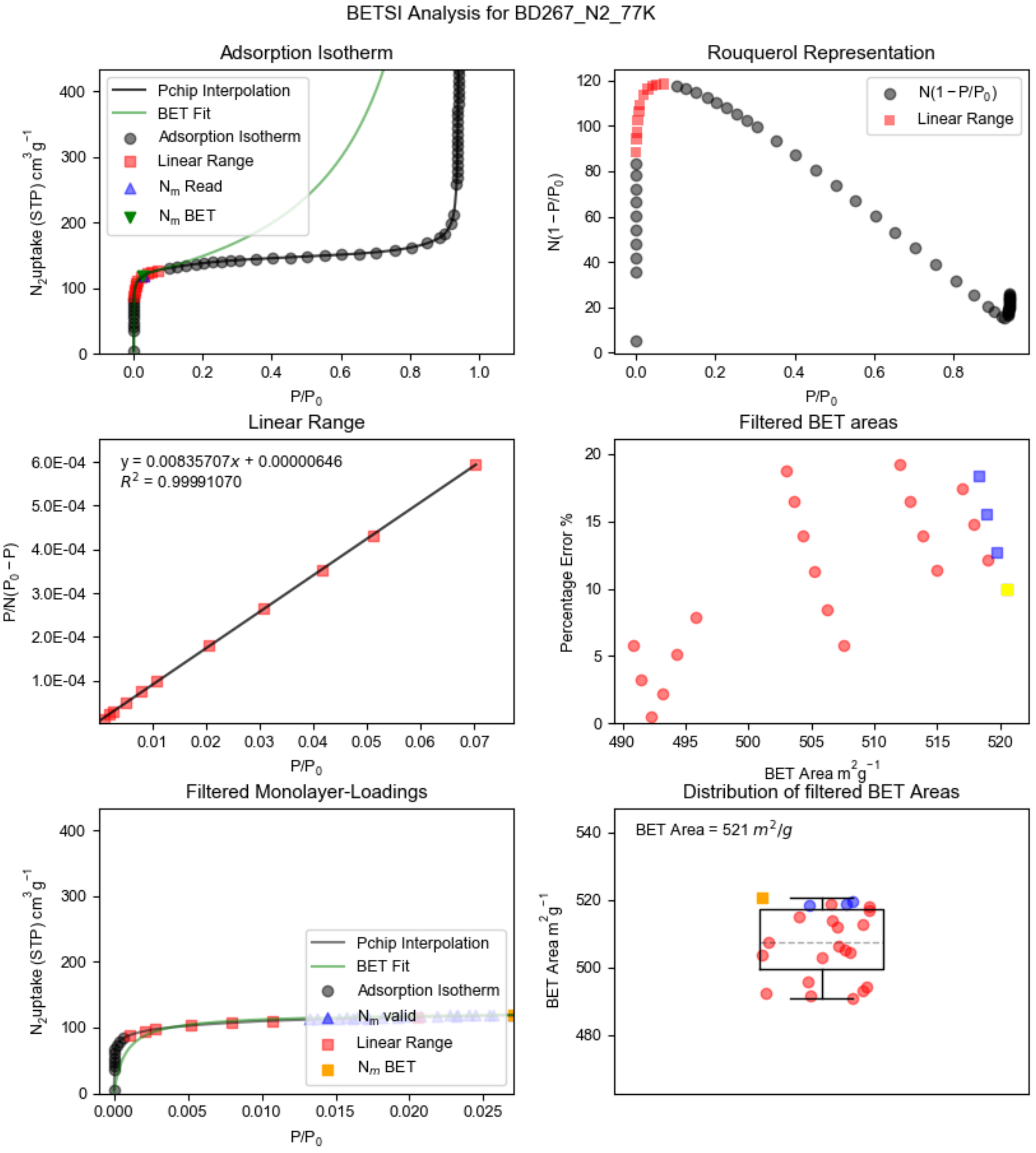


**Figure S32.** Plots obtained after running the "BET surface identification" (BETSI) program, as described in reference 17, implementing the extended Rouquerol criteria for unambiguous BET area assignment for **2a**. (Top left) $N_2$ adsorption isotherm of **2a** showing the best fitting region highlighted in red. (Middle left) Linear plot of the BET equation fitted by an ordinary least-squares regression applied to a particular region of the isotherm. (Bottom left) Focus on the 0 to 0.030 pressure range of the $N_2$ adsorption isotherm showing the $N_m$ (Read) and $N_m$ (BET), the monolayer loading calculated from BET. (Top right) $N(1-P/P_0)$ as a function of P showing the best fitting region in red where the Rouquerol representation monotically increase. If all criteria are met, the fitting is passed. (Middle right) All valid fitting results (acceptable BET areas output) plotted against the percentage error under the 4th Rouquerol criterion. The BETSI Optimal BET area (yellow) belongs to the isotherm knee group and has the lowest percentage error under the 4th Rouquerol criterion.

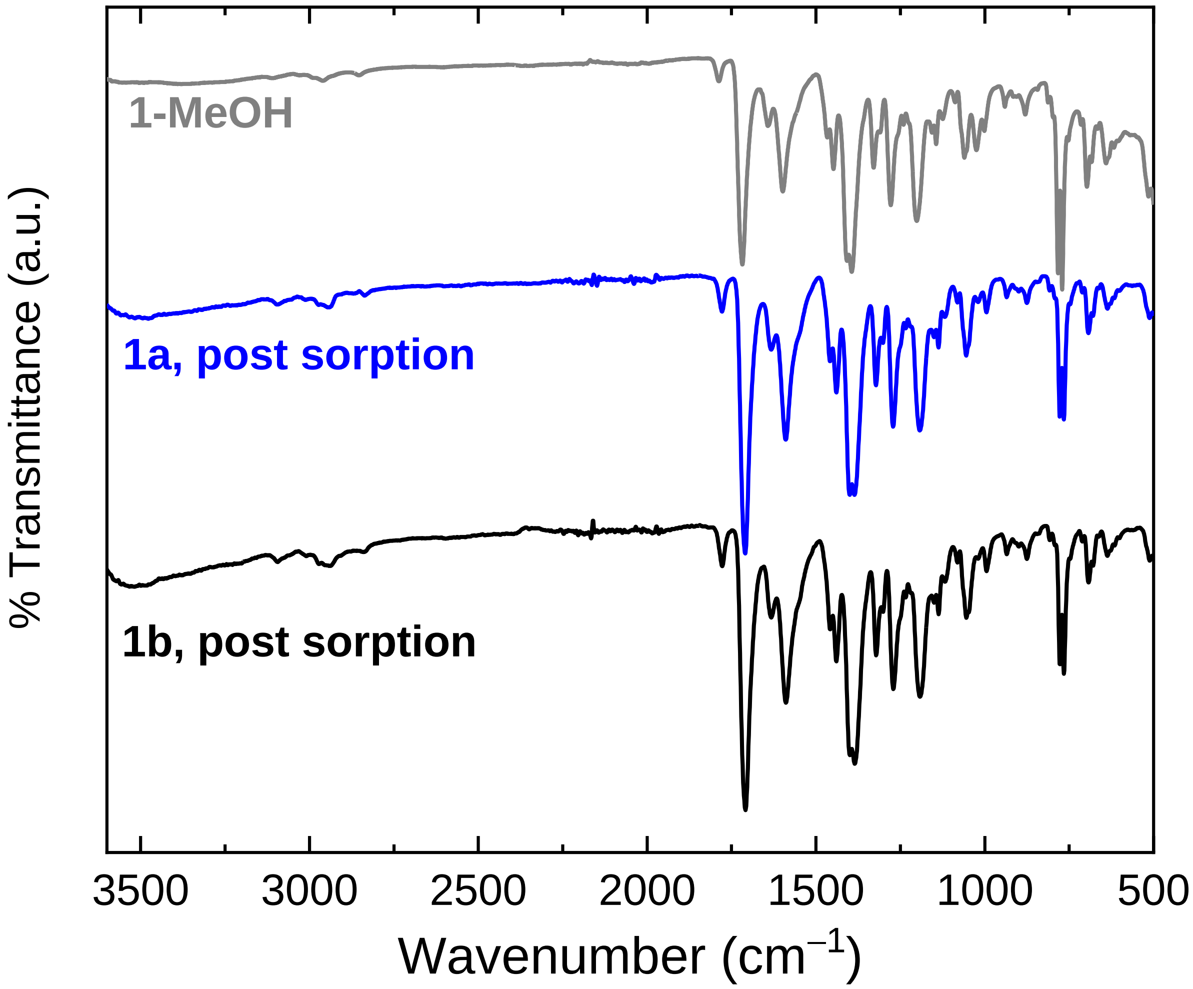


**Figure S33.** Comparison of the IR spectra for **1-MeOH**, and the sample recovered after activation and gas sorption measurements (**1a** and **1b**, activated at 120°C and 140°C, respectively).

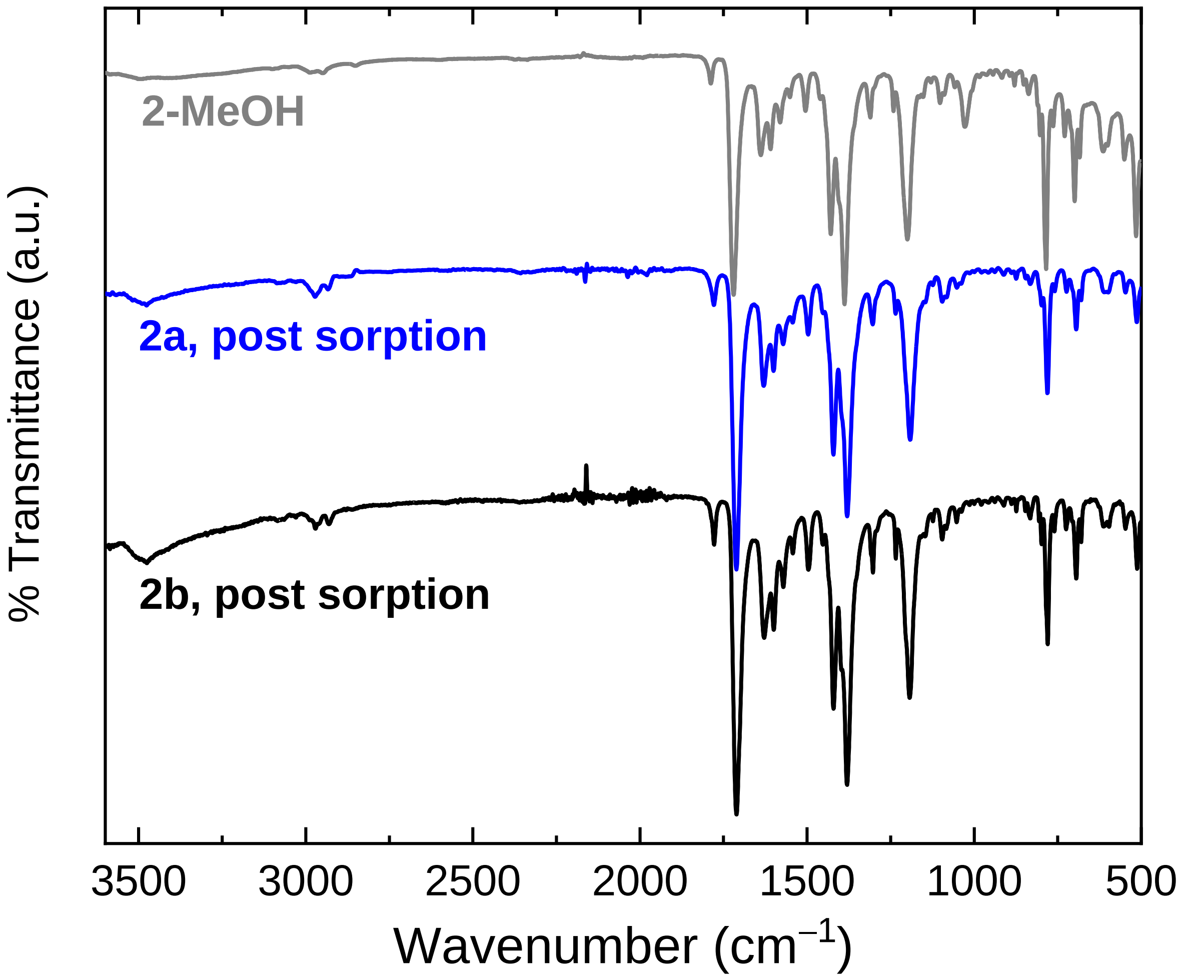


**Figure S34.** Comparison of the IR spectra for **2-MeOH**, and the sample recovered after activation and gas sorption measurements (**2a** and **2b**, activated at 120°C and 140°C, respectively).

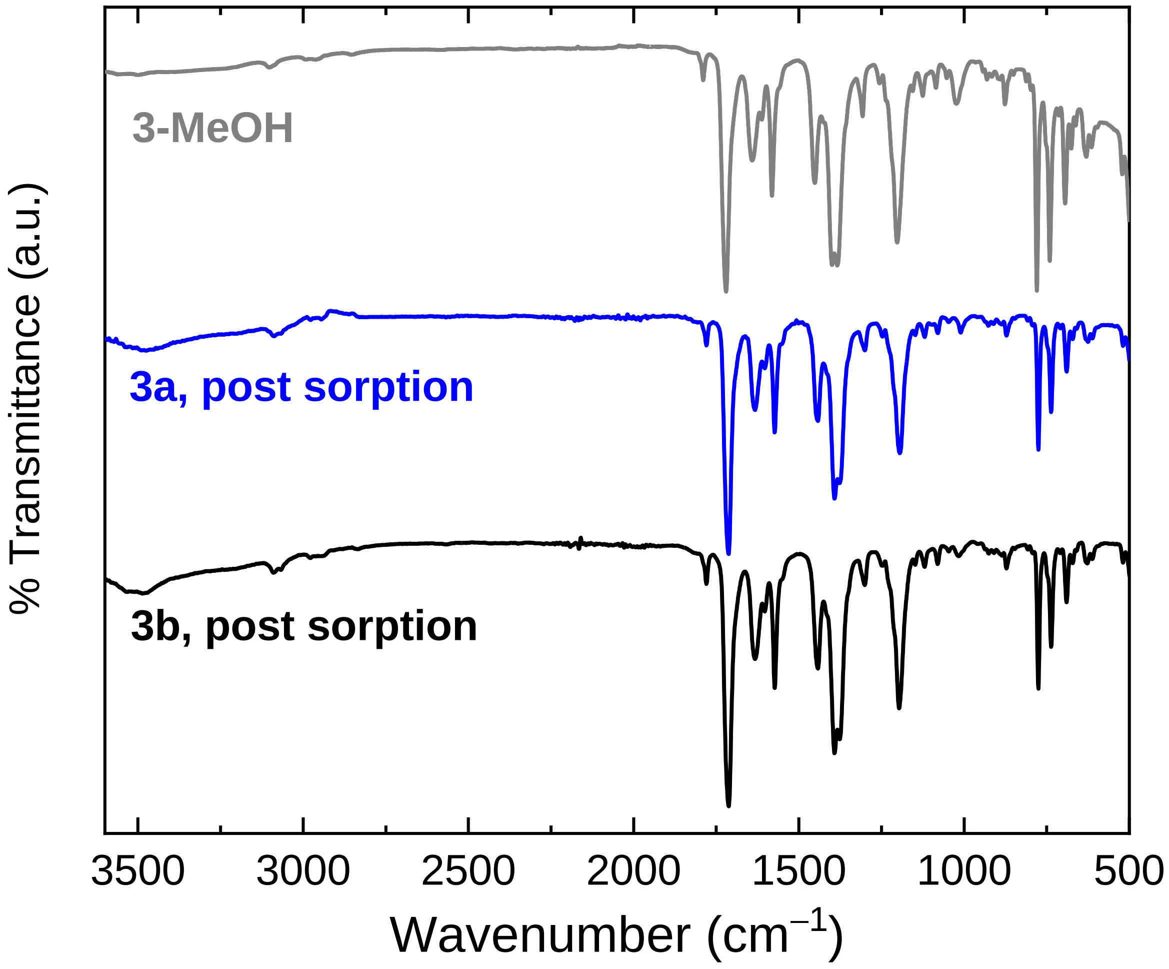


**Figure S35.** Comparison of the IR spectra for **3-MeOH**, and the sample recovered after activation and gas sorption measurements (**3a** and **3b**, activated at 120°C and 140°C, respectively).

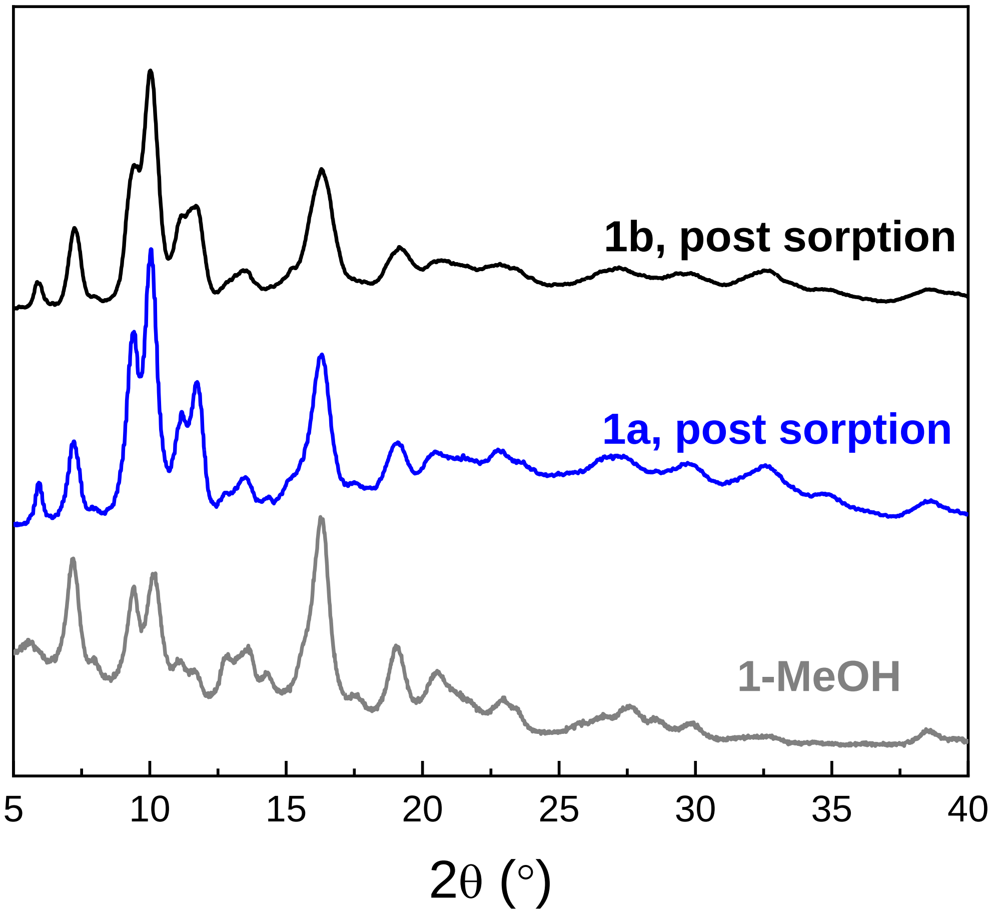


**Figure S36.** Comparison of the PXRD data for **1-MeOH**, and the sample recovered after activation and gas sorption measurements (**1a** and **1b**, activated at 120°C and 140°C, respectively).

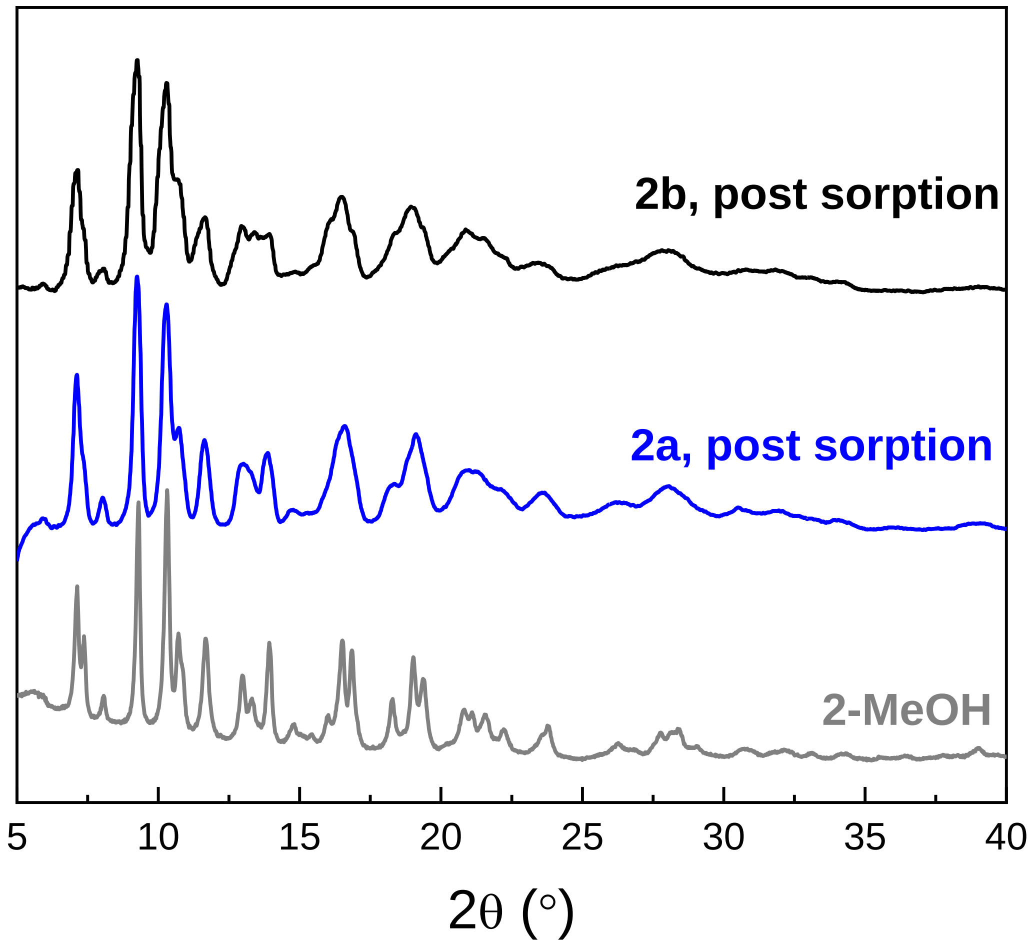


**Figure S37.** Comparison of the PXRD data for **2-MeOH**, and the sample recovered after activation and gas sorption measurements (**2a** and **2b**, activated at 120°C and 140°C, respectively).

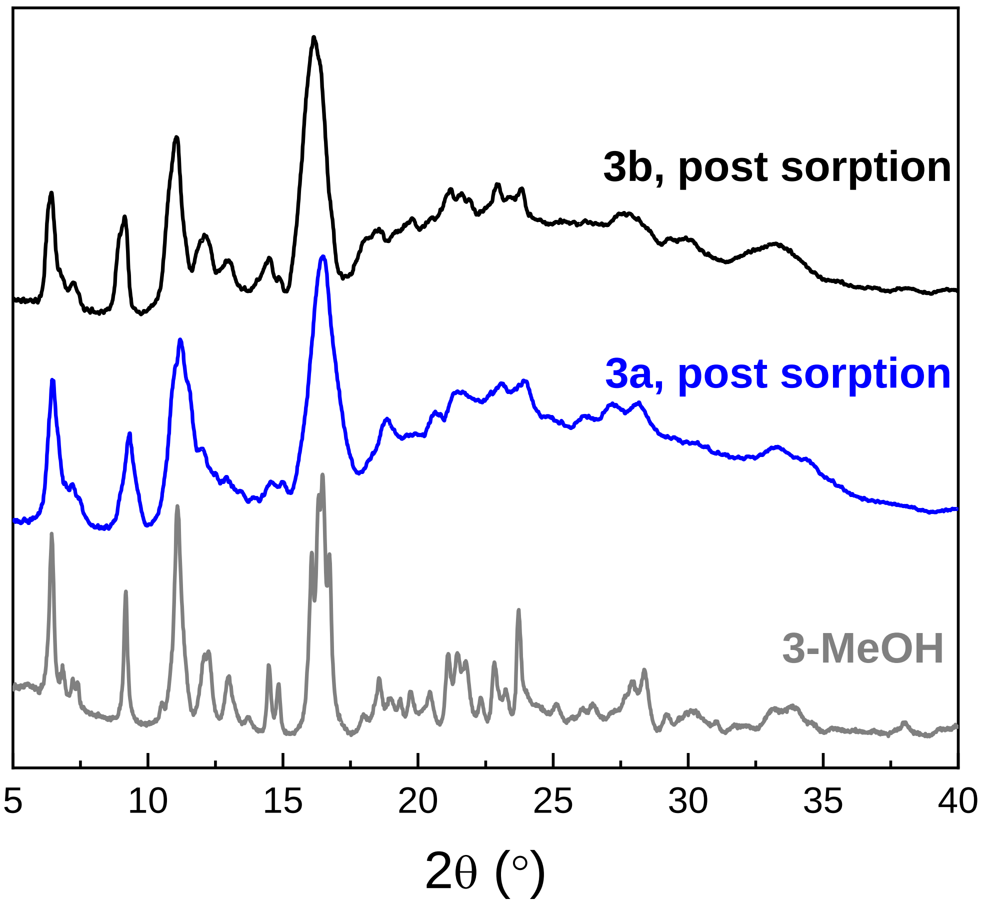


**Figure S38.** Comparison of the PXRD data for **3-MeOH**, and the sample recovered after activation and gas sorption measurements (**3a** and 3**b**, activated at 120°C and 140°C, respectively).

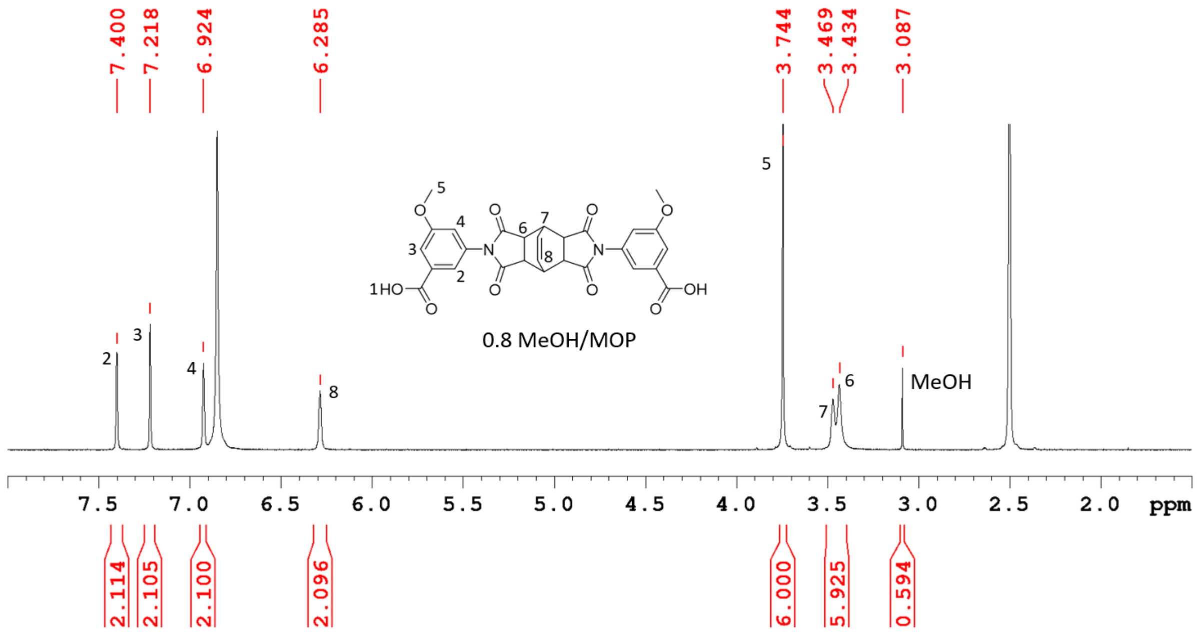


**Figure S39.** $^{1}$H-NMR spectrum of the digestion in DMSO/DCl of **1a** subsequent to gas sorption measurements.

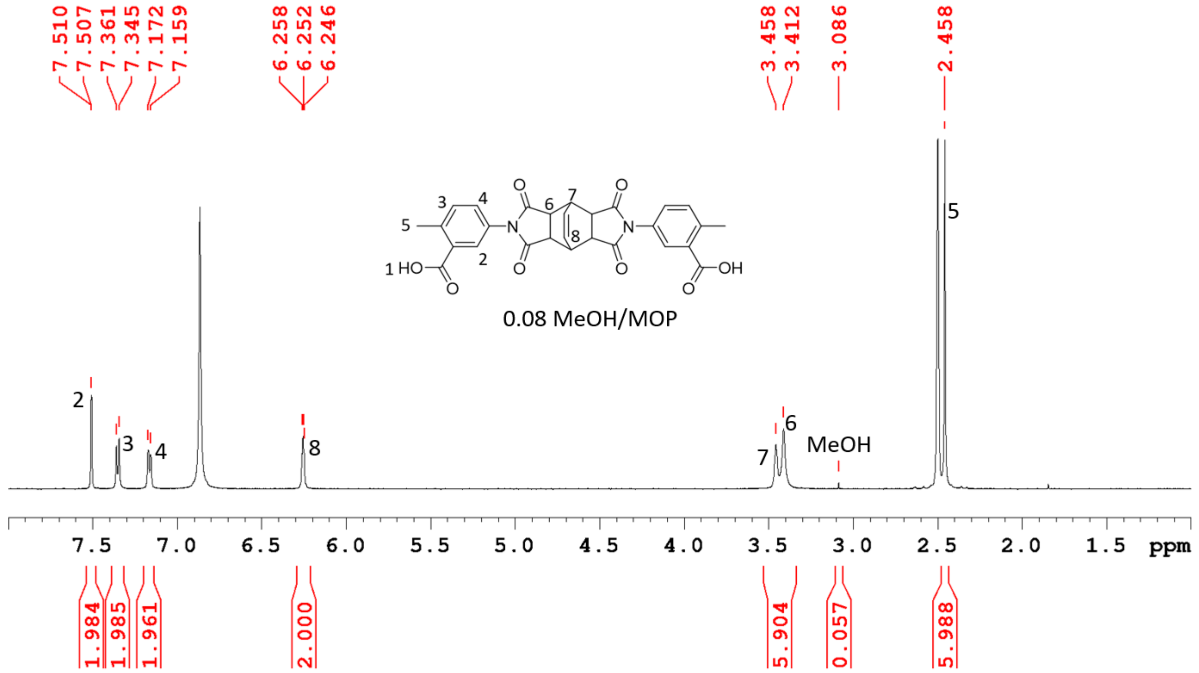


**Figure S40.** $^{1}$H-NMR spectrum of the digestion in DMSO/DCl of **2a** subsequent to gas sorption measurements.

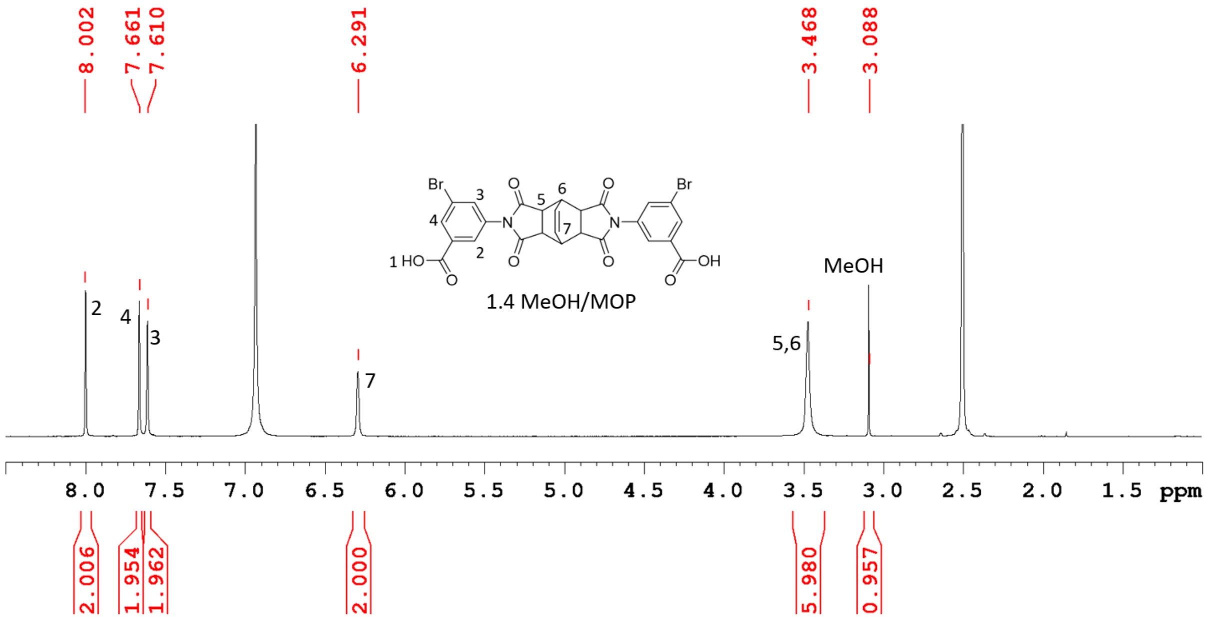


**Figure S41.** $^{1}$H-NMR spectrum of the digestion in DMSO/DCl of **3a** subsequent to gas sorption measurements.

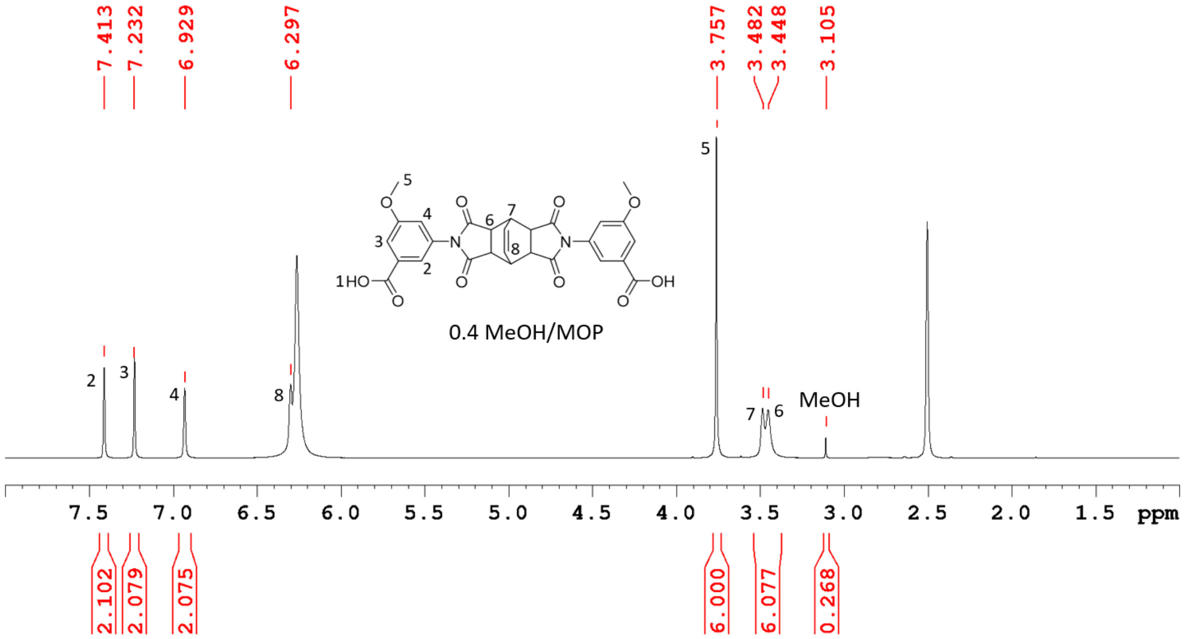


**Figure S42.** $^{1}$H-NMR spectrum of the digestion in DMSO/DCl of **1b** subsequent to gas sorption measurements.

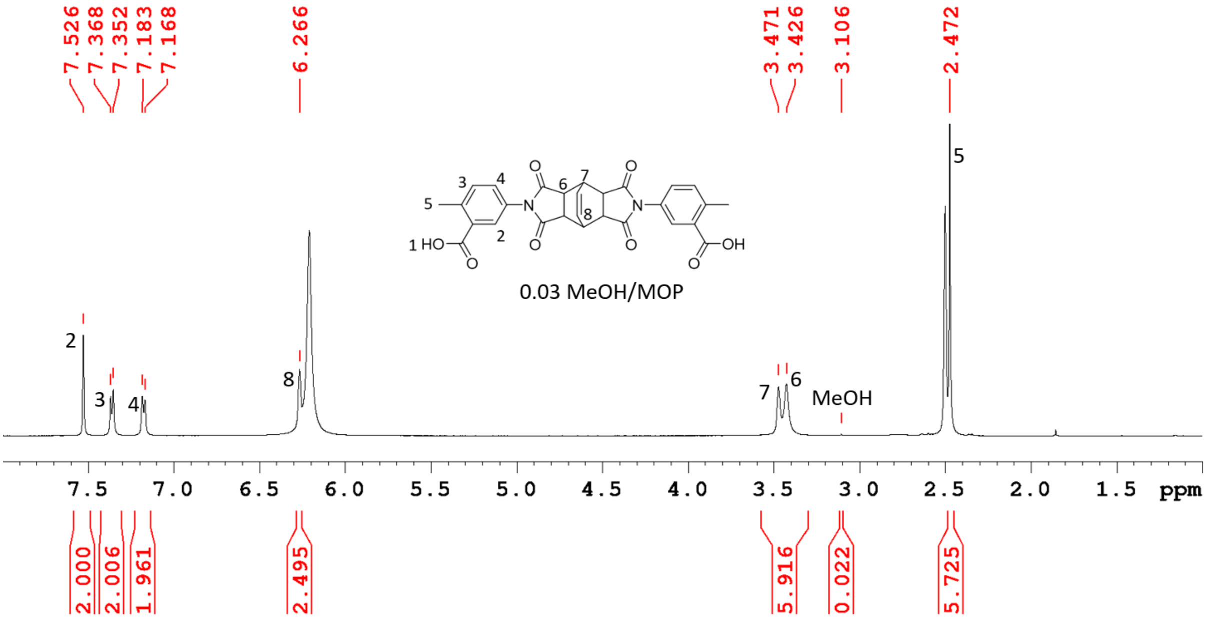


**Figure S43.** $^{1}$H-NMR spectrum of the digestion in DMSO/DCl of **2b** subsequent to gas sorption measurements.

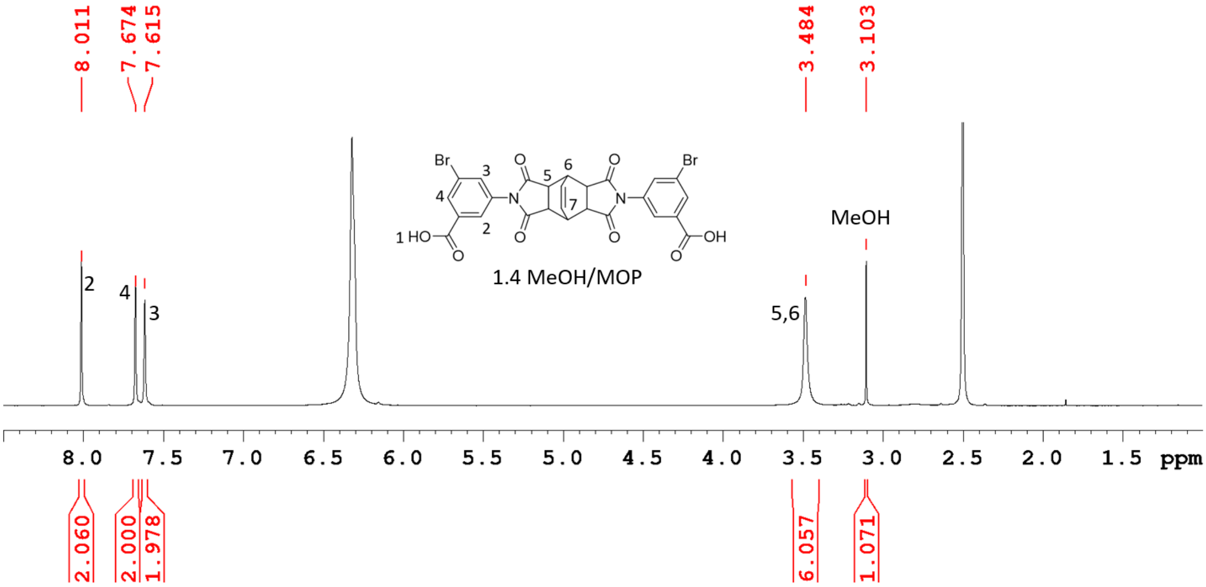


**Figure S44.** $^{1}$H-NMR spectrum of the digestion in DMSO/DCl of **3b** subsequent to gas sorption measurements.

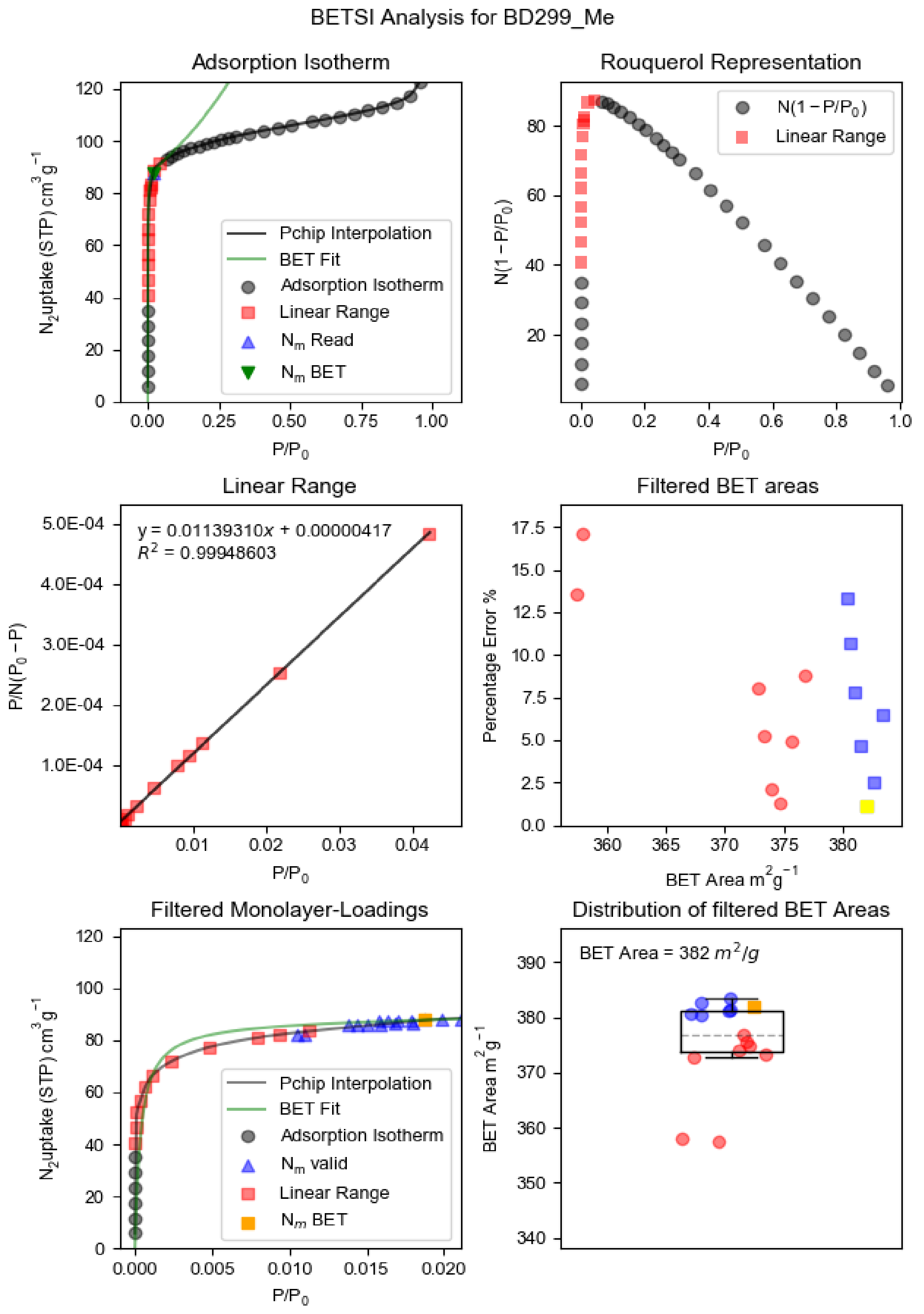


**Figure S45.** Plots obtained after running the "BET surface identification" (BETSI) program implementing the extended Rouquerol criteria for unambiguous BET area assignment for **2b**. See the caption for Figure S32 for a fuller description of the panels.

**Table S8.** Cartesian coordinates of the DFT calculated model cage.

| | | | |
|---|---|---|---|
| Cu | -6.955 | -0.0401 | -0.5775 |
| Cu | -4.466 | -0.0191 | -0.2672 |
| O | -6.6256 | 1.2059 | -2.052 |
| O | -9.1768 | 0.0036 | -0.0722 |
| O | -6.9458 | 1.494 | 0.7041 |
| O | 6.6233 | 1.5581 | 1.7683 |
| O | -4.8069 | 1.0688 | 1.2962 |
| O | 6.9436 | 1.2877 | -0.9847 |
| O | -3.5264 | 1.7776 | 5.8006 |
| O | 2.4134 | 6.2982 | 5.4294 |
| O | -4.5015 | 1.6004 | -1.3891 |
| O | 4.4162 | 1.0989 | 1.9099 |
| O | 4.7231 | 1.6341 | -0.7665 |
| O | 2.2757 | 1.7417 | 6.0698 |
| O | -2.7266 | 5.9426 | -1.9756 |
| N | 2.6613 | 3.9938 | 5.628 |
| N | -3.7825 | 4.0107 | 5.199 |
| O | -3.4746 | 6.3045 | 4.9681 |
| O | -2.3981 | 5.1746 | -6.5027 |
| O | -2.2219 | 0.1617 | -0.6763 |
| H | -2.1883 | -0.5221 | -1.3658 |
| H | -2.3331 | 0.991 | -1.1715 |
| N | 3.5719 | 5.4965 | -3.722 |
| N | -2.8662 | 5.4044 | -4.2355 |
| C | -4.919 | 3.859 | 4.3429 |
| O | 3.0662 | 6.0288 | -1.5143 |
| C | 3.9317 | 3.8578 | 4.9823 |
| C | 0.5974 | 3.4196 | 6.6582 |
| H | 0.5482 | 3.1703 | 7.7242 |
| C | 1.9211 | 2.8991 | 6.1086 |
| C | -4.887 | 2.8801 | 3.3513 |
| H | -4.0243 | 2.2388 | 3.2337 |
| C | -3.1445 | 2.9261 | 5.8275 |
| C | -5.9117 | 1.682 | 1.4167 |
| C | -6.031 | 4.6936 | 4.4813 |
| H | -6.0392 | 5.4634 | 5.2443 |
| C | 5.3179 | 2.7468 | 3.3423 |
| C | -5.9791 | 2.719 | 2.4937 |
| C | -5.3891 | 2.8939 | -3.1544 |
| O | 3.4863 | 5.2719 | -6.0349 |
| C | -7.0981 | 3.5497 | 2.6321 |
| C | 5.4676 | 1.7251 | 2.2596 |
| C | 6.398 | 3.5563 | 3.7159 |
| C | 5.004 | 4.6748 | 5.3507 |
| C | -3.9316 | -3.8578 | -4.9823 |
| C | -0.5974 | -3.4196 | -6.6582 |
| H | -0.5481 | -3.1703 | -7.7242 |
| C | -1.921 | -2.8991 | -6.1086 |
| C | 4.887 | -2.8801 | -3.3513 |
| H | 4.0244 | -2.2388 | -3.2337 |
| C | 3.1445 | -2.9261 | -5.8275 |
| C | 5.9117 | -1.682 | -1.4167 |
| C | 6.031 | -4.6936 | -4.4813 |
| H | 6.0393 | -5.4634 | -5.2443 |
| C | -5.3178 | -2.7468 | -3.3423 |
| C | 5.9792 | -2.719 | -2.4937 |
| C | 5.3891 | -2.8939 | 3.1544 |
| O | -3.4863 | -5.2719 | 6.0349 |
| C | 7.0981 | -3.5497 | -2.6321 |
| C | -5.4676 | -1.7251 | -2.2596 |
| C | -6.398 | -3.5563 | -3.7159 |
| C | -5.004 | -4.6747 | -5.3507 |
| H | -4.8733 | -5.4247 | -6.1222 |
| C | -0.6212 | -4.9409 | -6.4023 |
| H | -0.5538 | -5.528 | -7.3251 |
| C | -4.0822 | -2.9047 | -3.9766 |
| H | -3.2516 | -2.2764 | -3.688 |
| C | 5.5209 | -1.8215 | 2.119 |
| H | 4.8734 | 5.4247 | 6.1222 |
| C | 0.6213 | 4.941 | 6.4023 |
| H | 0.5538 | 5.528 | 7.3251 |
| C | 4.0822 | 2.9047 | 3.9767 |
| H | 3.2516 | 2.2764 | 3.688 |
| C | -5.5209 | 1.8215 | -2.119 |
| C | -7.1163 | 4.5337 | 3.6186 |
| H | -7.9792 | 5.1855 | 3.718 |
| C | 1.9816 | 5.2184 | 5.7663 |
| C | 5.862 | 1.8906 | -1.2652 |
| C | 5.9098 | 2.9953 | -2.2736 |
| C | 4.7499 | 3.7321 | -2.5319 |
| H | 3.844 | 3.5 | -1.9889 |
| C | -0.5586 | 5.334 | 5.4581 |
| H | -0.5347 | 6.4018 | 5.2345 |
| C | -0.6114 | 2.7749 | 5.9172 |
| H | -0.6322 | 1.696 | 6.0783 |
| C | -4.2192 | 3.657 | -3.2122 |
| H | -3.4348 | 3.4841 | -2.4889 |
| C | -1.8866 | 3.4442 | 6.5146 |
| H | -1.9548 | 3.2093 | 7.5826 |
| C | -2.2693 | 5.9743 | -3.0963 |
| C | -0.4868 | 4.4697 | 4.2174 |
| H | -0.4235 | 4.9111 | 3.2275 |
| C | -0.9472 | 6.6081 | -3.5141 |
| H | -0.9908 | 7.6775 | -3.2794 |
| C | 6.2334 | 4.5193 | 4.7093 |
| H | 7.0679 | 5.1543 | 4.9916 |
| C | -1.8625 | 4.9612 | 6.2331 |
| H | -1.9249 | 5.5632 | 7.1466 |
| C | -4.0762 | 4.6411 | -4.1892 |
| C | -0.5196 | 3.1516 | 4.4534 |
| H | -0.4884 | 2.3928 | 3.6772 |
| C | -6.4138 | 3.1222 | -4.0815 |
| C | 0.4141 | 4.2499 | -4.4379 |
| H | 0.4709 | 3.2567 | -4.8728 |
| C | -3.1129 | 5.232 | 5.3982 |
| C | 0.3057 | 4.4923 | -3.1249 |
| H | 0.2669 | 3.719 | -2.3635 |
| C | 0.2507 | 5.9601 | -2.7568 |
| H | 0.1625 | 6.1259 | -1.682 |
| C | 1.5329 | 6.6474 | -3.3162 |
| H | 1.5067 | 7.7169 | -3.0791 |
| C | -2.0947 | 5.5735 | -5.4005 |
| C | -6.2334 | -4.5193 | -4.7093 |
| H | -7.0679 | -5.1543 | -4.9916 |
| C | 1.8625 | -4.9612 | -6.2331 |
| H | 1.9249 | -5.5632 | -7.1467 |
| C | 4.0762 | -4.6411 | 4.1892 |
| C | 0.5197 | -3.1515 | -4.4534 |
| H | 0.4884 | -2.3928 | -3.6772 |
| C | 6.4138 | -3.1222 | 4.0815 |
| C | -0.4141 | -4.2499 | 4.4379 |
| H | -0.471 | -3.2567 | 4.8728 |
| C | 3.1129 | -5.232 | -5.3982 |
| C | -0.3057 | -4.4923 | 3.1249 |
| H | -0.2669 | -3.719 | 2.3635 |
| C | -0.2507 | -5.9601 | 2.7568 |
| H | -0.1625 | -6.1259 | 1.682 |
| C | -1.5329 | -6.6474 | 3.3162 |
| H | -1.5067 | -7.7169 | 3.0791 |
| C | 2.0947 | -5.5735 | 5.4005 |
| C | -1.6585 | -6.3724 | 4.8296 |
| H | -1.6892 | -7.2906 | 5.4268 |
| C | -4.7712 | -4.7547 | 3.4782 |
| C | 0.8229 | -6.3321 | 5.0269 |
| H | 0.7883 | -7.2502 | 5.624 |
| C | -2.9961 | -5.6534 | 4.9957 |
| C | 1.6585 | 6.3724 | -4.8296 |
| H | 1.6892 | 7.2906 | -5.4268 |
| C | 4.7712 | 4.7547 | -3.4782 |
| C | -0.823 | 6.3321 | -5.0269 |
| H | -0.7883 | 7.2502 | -5.624 |
| C | 2.9961 | 5.6534 | -4.9957 |
| C | 7.0858 | 3.2869 | -2.9756 |
| C | 2.789 | 6.0492 | -2.6925 |
| C | -5.0926 | 4.8661 | -5.1218 |
| H | -4.9668 | 5.622 | -5.8882 |
| C | -6.2577 | 4.1005 | -5.0613 |
| H | -7.0475 | 4.271 | -5.7868 |
| C | 0.4614 | 5.4894 | -5.3058 |
| H | 0.5496 | 5.2607 | -6.3691 |
| C | 5.9397 | 5.0415 | -4.1884 |
| H | 5.9409 | 5.8277 | -4.9348 |
| C | 7.0935 | 4.2996 | -3.9326 |
| H | 8.003 | 4.5167 | -4.4849 |
| Cu | 6.955 | 0.0401 | 0.5775 |
| Cu | 4.466 | 0.0191 | 0.2672 |
| O | 6.6256 | -1.2059 | 2.052 |
| O | 9.1769 | -0.0036 | 0.0722 |
| O | 6.9458 | -1.494 | -0.7041 |
| O | -6.6233 | -1.5581 | -1.7683 |
| O | 4.8069 | -1.0688 | -1.2962 |
| O | -6.9436 | -1.2877 | 0.9847 |
| O | 3.5264 | -1.7776 | -5.8006 |
| O | -2.4134 | -6.2982 | -5.4294 |
| O | 4.5015 | -1.6004 | 1.3891 |
| O | -4.4162 | -1.0989 | -1.9099 |
| O | -4.7231 | -1.6341 | 0.7665 |
| O | -2.2757 | -1.7417 | -6.0698 |
| O | 2.7266 | -5.9426 | 1.9756 |
| N | -2.6613 | -3.9938 | -5.628 |
| N | 3.7825 | -4.0107 | -5.199 |
| O | 3.4747 | -6.3044 | -4.9681 |
| O | 2.3981 | -5.1746 | 6.5027 |
| O | 2.2219 | -0.1617 | 0.6764 |
| H | 2.1883 | 0.5221 | 1.3658 |
| H | 2.3331 | -0.991 | 1.1716 |
| N | -3.5719 | -5.4965 | 3.722 |
| N | 2.8661 | -5.4044 | 4.2355 |
| C | 4.919 | -3.859 | -4.3429 |
| O | -3.0663 | -6.0288 | 1.5143 |
| H | -9.1193 | -0.7266 | 0.5667 |
| H | -9.0935 | 0.8033 | 0.4746 |
| H | 9.1193 | 0.7266 | -0.5667 |
| H | 9.0935 | -0.8033 | -0.4746 |

| | | | | | | | |
|---|---|---|---|---|---|---|---|
| C | 7.1163 | -4.5337 | -3.6186 | C | -7.0858 | -3.2869 | 2.9756 |
| H | 7.9793 | -5.1854 | -3.718 | C | -2.7891 | -6.0492 | 2.6925 |
| C | -1.9816 | -5.2184 | -5.7663 | C | 5.0926 | -4.8661 | 5.1218 |
| C | -5.862 | -1.8906 | 1.2652 | H | 4.9668 | -5.622 | 5.8882 |
| C | -5.9099 | -2.9953 | 2.2736 | C | 6.2577 | -4.1005 | 5.0613 |
| C | -4.7499 | -3.7321 | 2.5319 | H | 7.0475 | -4.2711 | 5.7868 |
| H | -3.844 | -3.5 | 1.9889 | C | -0.4614 | -5.4894 | 5.3058 |
| C | 0.5586 | -5.334 | -5.4581 | H | -0.5497 | -5.2607 | 6.3691 |
| H | 0.5347 | -6.4018 | -5.2345 | C | -5.9397 | -5.0415 | 4.1885 |
| C | 0.6114 | -2.7749 | -5.9172 | H | -5.9409 | -5.8277 | 4.9348 |
| H | 0.6322 | -1.696 | -6.0783 | C | -7.0935 | -4.2996 | 3.9326 |
| C | 4.2192 | -3.657 | 3.2122 | H | -8.003 | -4.5167 | 4.4849 |
| H | 3.4348 | -3.4841 | 2.4889 | H | -7.9358 | 3.4269 | 1.9551 |
| C | 1.8866 | -3.4442 | -6.5146 | H | 7.3518 | 3.4238 | 3.2183 |
| H | 1.9549 | -3.2093 | -7.5826 | H | -7.3518 | -3.4238 | -3.2183 |
| C | 2.2692 | -5.9743 | 3.0963 | H | 7.9358 | -3.4269 | -1.9551 |
| C | 0.4868 | -4.4697 | -4.2174 | H | -7.3159 | 2.5237 | -4.0273 |
| H | 0.4235 | -4.9111 | -3.2275 | H | -7.9806 | -2.7102 | 2.7719 |
| C | 0.9472 | -6.6081 | 3.5141 | H | 7.3159 | -2.5237 | 4.0273 |
| H | 0.9908 | -7.6775 | 3.2794 | H | 7.9806 | 2.7102 | -2.7719 |
